# Analysis of miRNAs and their target genes in five *Melilotus albus* NILs with different coumarin content

**DOI:** 10.1038/s41598-018-32153-3

**Published:** 2018-09-20

**Authors:** Fan Wu, Kai Luo, Zhuanzhuan Yan, Daiyu Zhang, Qi Yan, Yufei Zhang, Xianfeng Yi, Jiyu Zhang

**Affiliations:** 1 State Key Laboratory of Grassland Agro-ecosystems, Key Laboratory of Grassland Livestock Industry Innovation, Ministry of Agriculture and Rural Affairs, College of Pastoral Agriculture Science and Technology, Lanzhou University, Lanzhou, 730020 P.R. China; 2 Guangxi Institute of Animal Sciences, Nanning, 530001 P.R. China

## Abstract

MicroRNAs (miRNAs) exhibit diverse and important roles in regulation of various biological processes at the post-transcriptional level in plants. In this study, *Melilotus albus* miRNA and their target genes were elucidated from five *M*. *albus* near-isogenic lines which differ in coumarin content to construct small RNA libraries through high-throughput sequencing. A total of 417 known miRNAs and 76 novel miRNAs were identified in *M*. *albus*. In addition, 4155 different target genes for 114 known miRNA families and 14 target genes for 2 novel miRNAs were identified in *M*. *albus*. Moreover, mtr-miR5248 and mtr-miR7701-5p target c35498_g3 and gma-miR396a-3p target c37211_g1 involved in coumarin biosynthesis were identified by using the differential expression of the miRNAs and their target genes correlation analysis. The abundance of miRNAs and potential target genes were validated by qRT-PCR analysis. We also found that there were both positive and negative expression changing patterns between miRNAs and their related target genes. Our first and preliminary study of miRNAs will contribute to our understanding of the functions and molecular regulatory mechanisms of miRNAs and their target genes, and provide information on regulating the complex coumarin pathway in *M*. *albus* for future research.

## Introduction

*Melilotus* is a Leguminosae and an annual or biennial herbal. This genus comprises nearly 19 species globally, among which mainly *M*. *albus* and *M*. *officinalis* are leveraged in production and research studies. Members of the *Melilotus* genus are well known for theirs high seed yields and high tolerance in extreme environment such as drought, cold and high salinity^1,2^. It is an important forage green manure crop in combination of agriculture and animal husbandry in northern China, and it can be used as a crop fertilizer. People have become increasingly concerned about its medicinal value with its variety of biological activity of coumarin, flavone, saponin. As a cheap, abundant Chinese herbal medicine plant resource, *Melilotus* is worthy of further development for its vast market value.

Coumarins are a major group of natural plant products derived from the phenylpropanoid pathway which are found in different species of plants in nature, and they can be classified into four categories: simple coumarins, pyronesubstituted coumarins, furanocoumarins, and pyranocoumarins^3–6^. A large number of studies focusing on the therapeutic and pharmacologic properties of coumarins have supported their therapeutic roles. Previous studies have found that coumarins have many biological activities, such as anti-HIV, anti-tumour, anti-hypertension, anti-oxidation, anti-inflammation, anti-arrhythmia and anti-osteoporosis, in addition to assuaging pain, preventing asthma and antisepsis, and possessing other pharmacological activities^7^. However, coumarin widely occurs in *Melilotus*, and it may be a major limiting factor for the use of forage legumes. The coumarin content ranges from 0.08% to 1.39% of the dry matter in 15 *Melilotus* species^8^. Coumarin has been associated with dicoumarol production upon spoilage by fungi in *M*. *albus*^9^. It is impermissible for grazing animals with high concentration of dicoumarol in forage or conserved fodder^10^. β-glucosidase activity in *Melilotus* is related to a bitter taste which is reduced the palatability. Such comparison of contrasting coumarin contents at different β-glucosidase activity levels in *M*. *albus* might help in a further understanding of coumarin biosynthesis and its regulatory network.

MicroRNAs (miRNAs) are a class of non-coding endogenous small RNAs with 20 to 24 nt that exist widely in diversified plants^11^. In previous studies, much evidence has indicated that miRNAs diverse and important roles in regulating such as leaf, stem, root and flower development, the phase switch from vegetative growth to reproductive growth, and abiotic and biotic stress responses in plants^12^. miRNAs regulate gene expression at the post-transcriptional level and not directly regulate plant growth and development^11,13^. It was discovered that five conserved miRNAs (Agr-miR159, Agr-miR164, Agr-miR166, Agr-miR396, and Agr-miR408) were regulated during celery leaf development, and they were expressed in the petioles and leaf blades of ‘Ventura’ at the three stages related to leaf development^14^. The results of *Halostachys caspica* research suggested that miRNAs play an important role in plant salt stress tolerance because of miRNAs and their target genes were responsive to high salt stress, and a negative expression correlation between miRNAs and their target genes existed in *H*. *caspica*^15^. Many miRNAs were found that were changed significantly in the leaf under 300 mM NaCl stress in *Populus euphratica* by deep sequencing^16^. All of the evidence indicated that miRNA regulation plays an essential role as a regulatory mechanism to the response. However, miRNAs have remained unknown in *Melilotus* until now. Therefore, it is necessary to investigate *Melilotus* miRNAs and their target genes.

Our previous results showed that the interspecific relationships within the *Melilotus* genus based on the phylogenetic tree are clearly monophyletic in the legume family^17^. We also analysed the genetic diversity among 50 accessions of 18 *Melilotus* species using SSR markers^18^. In the breeding programme of low-coumarin *Melilotus* species, a preliminary evaluation of agronomy and the quality traits of 19 *Melilotus* accessions showed that coumarin content could vary from 0.16–1.02%^19^. A key objective in the breeding programme of *M*. *albus* is to improve the dry matter yield and to decrease the coumarin content. However, knowledge at the genomics level in *Melilotus* and the coumarin pathway remain unknown, and there are no reports about the roles of miRNAs and their target genes in *Melilotus*.

In this study, we used five near-isogenic lines (NILs) of *M*. *albus* to perform sRNA and high-throughput sequencing for the first time, resulting in 417 known miRNAs and 76 novel miRNAs, as well as 4169 miRNA target genes of *M*. *albus*. In addition, Gene Ontology (GO) and Kyoto Encyclopedia of Genes and Genomes (KEGG) analysis revealed the involvement of *M*. *albus* miRNA target genes in plant hormone signal transduction, the regulation of transcription and biological processes, which will help further investigation of the biological functions and regulatory mechanisms of miRNAs and their target genes and the understanding of coumarin biosynthesis and its regulatory network in *M*. *albus*.

## Results

### Analysis of small RNAs in *M*. *albus*

A total of 23.8, 24.7, 23.5, 24.4 and 24.2 million reads were generated from N46, N47, N48, N49 and RPh, the five genotypes of *M*. *albus*, respectively. The Q20 values were greater than 98.81% and the GC percentages were 48.92%, 48.66%, 48.94%, 48.86% and 48.62% for the five genotypes (Additional file 1: Table S1). The sequencing data are available at the NCBI number Bioproject with accession number PRJNA356361 with biosample accession number SRS1842864, SRS1842865, SRS1842878, SRS1842884 and SRS1842885 for N46, N47, N48, N49 and RPh, respectively. After filtering out the reads with N%> 10%, low quality, 5′ adapter contaminants, 3′ adapter null or insert null and poly A/T/G/C, a total of 23.3, 24.2, 23.1, 23.9 and 23.7 million high quality clean reads were obtained, respectively (Additional file 2: Table S2). The length of the small RNAs was between 18 nt and 30 nt, and the sequences of small RNAs with 21, 22, 23 and 24 nt had the highest abundance (Additional file 3: Fig. S1). The length distributions of small RNAs were similar for five genotypes of *M*. *albus*. The most abundant small RNAs were found in 24 nt, representing 44.26%, 44.36%, 40.78%, 42.95% and 43.19% of the small RNAs in the five genotypes.

The small RNAs were further mapped to known miRNA, rRNA, tRNA, snRNA, snoRNA, repeat, novel miRNA, TAS and other unannotated RNAs by performing BLAST searches against the Rfam database (Table 1). The majority of unique sRNAs were mapped to rRNAs and other unannotated RNAs which were not included known miRNA, ncRNA, repeat, novel miRNA and TAS. The unique sRNAs of 2217 (0.14%), 2538 (0.14%), 2248 (0.14%), 2099 (0.12%) and 2427 (0.13%) were annotated as known miRNAs in N46, N47, N48, N49 and RPh, respectively. Additionally, in total, 1295 (0.08%), 1552 (0.09%), 1440 (0.09%), 1322 (0.07%) and 1565 (0.08%) unique sRNAs were considered as novel miRNAs in the five genotypes of *M*. *albus*.

**Table 1:** Distribution of small RNAs among different types in *M*. *albus*.

Types	Total sRNAs					Unique sRNAs				
	N46	N47	N48	N49	RPh	N46	N47	N48	N49	RPh
total	7313787 (100%)	8508516 (100%)	8163350 (100%)	8826814 (100%)	8642278 (100%)	1637015 (100%)	1792088 (100%)	1586989 (100%)	1765914 (100%)	1926216 (100%)
known miRNA	728682 (9.96%)	1028910 (12.09%)	904832 (11.08%)	720146 (8.16%)	923123 (10.68%)	2217 (0.14%)	2538 (0.14%)	2248 (0.14%)	2099 (0.12%)	2427 (0.13%)
rRNA	308232 (4.21%)	286785 (3.37%)	369614 (4.53%)	523157 (5.93%)	304582 (3.52%)	29940 (1.83%)	28064 (1.57%)	31052 (1.96%)	33094 (1.87%)	27797 (1.44%)
tRNA	1 (0%)	0 (0%)	0 (0%)	0 (0%)	1 (0%)	1 (0%)	0 (0%)	0 (0%)	0 (0%)	1 (0%)
snRNA	2227 (0.03%)	2439 (0.03%)	2035 (0.02%)	2115 (0.02%)	2803 (0.03%)	994 (0.06%)	1085 (0.06%)	964 (0.06%)	1021 (0.06%)	1344 (0.07%)
snoRNA	6773 (0.09%)	7134 (0.08%)	6050 (0.07%)	6783 (0.08%)	8260 (0.1%)	1301 (0.08%)	1382 (0.08%)	1208 (0.08%)	1326 (0.08%)	1503 (0.08%)
repeat	22383 (0.31%)	28708 (0.34%)	23994 (0.29%)	25530 (0.29%)	28055 (0.32%)	7752 (0.47%)	9122 (0.51%)	8135 (0.51%)	9228 (0.52%)	9453 (0.49%)
novel miRNA	48166 (0.66%)	71178 (0.84%)	66860 (0.82%)	45880 (0.52%)	65262 (0.76%)	1295 (0.08%)	1552 (0.09%)	1440 (0.09%)	1322 (0.07%)	1565 (0.08%)
TAS	87310 (1.19%)	118079 (1.39%)	120870 (1.48%)	94845 (1.07%)	157397 (1.82%)	9285 (0.57%)	11009 (0.61%)	9743 (0.61%)	8876 (0.5%)	12419 (0.64%)
others	6110013 (83.54%)	6965283 (81.86%)	6669095 (81.7%)	7408358 (83.93%)	7152795 (82.77%)	1584230 (96.78%)	1737336 (96.94%)	1532199 (96.55%)	1708948 (96.77%)	1869707 (97.07%)

The total sRNAs were annotated as known miRNA, rRNA, tRNA, snRNA, snoRNA, repeat, novel miRNA, TAS and others.

### Identification of known miRNAs

A total of 417 known miRNAs were identified from five *M*. *albus* genotypes based on all unique plant known miRNAs libraries (Table 2). These known miRNAs belonged to 114 miRNA families and they were found that the distributions were similar between the five genotypes. The analysis of transcripts per million (TPM) value for the known miRNA indicated that the expression frequency varied significantly from 0 to 298447. For example, TPM values of various members in the miR156 family were tremendously different from each other, ranging from 0 to 19676. Several families such as miR156, miR159, miR166, miR171, miR172 and miR396, were relatively abundant, whereas some families were not. The largest family was miR156 with 33 members (Additional file 4: Table S3). The base bias on the first nucleotide of miRNA and the miRNA nucleotide bias at each position are shown in Additional file 5: Fig. S2. The result revealed that these miRNAs started with a 5′-U, which is consistent with typical miRNA sequence patterns based on miRBase 19.0 (http://www.mirbase.org/).

**Table 2:** Distribution of known and novel miRNAs of *M*. *albus* in five genotypes.

Types	Total	N46	N47	N48	N49	RPh
known miRNAs	417	329	341	330	322	341
novel miRNAs	76	72	75	75	72	75

### Identification of novel miRNAs

In total, 76 novel miRNA were identified among the five genotypes in *M*. *albus*. Among these novel miRNAs, 72, 75, 75, 72 and 75 were expressed in N46, N47, N48, N49 and RPh, respectively (Table 2). Similarly to the known miRNAs, the expression of the novel miRNAs also varied largely between the five libraries. Most of the novel predicted miRNAs had relatively highly abundances, and they were mostly observed in five genotypes. However, novel_146 was only observed in RPh (Additional file 6: Table S4). The novel miRNA sequences ranged from 18 to 24 nt in length, and 21 nt was the most frequent length. The length of the precursor miRNA sequences varied from 43 to 291 nt, and the minimum free energy (MFE) of the identified miRNA precursors varied from −149.6 to −12.3 kcal/mol with an average of approximately −44.7 kcal/mol, indicating high stability in the hairpin structures (Additional file 7: Table S5). Several secondary structures of the novel miRNA precursors are shown in Additional file 8: Fig. S3.

### Analysis of transcriptome sequences in *M*. *albus*

Approximately 32.9, 31.2, 32.5, 31.5 and 35.4 million raw reads were collected from the five genotypes of N46, N47, N48, N49 and RPh transcriptome libraries, respectively. All data from the raw sequence reads were deposited in the NCBI Sequence Read Archive (SRA, http://www.ncbi.nlm.nih.gov/Traces/sra) under the BioProject accession PRJNA331091. To ensure the reliability of the libraries, we performed quality controls and obtained approximately 30.5, 28.8, 30.1, 29.0 and 32.6 million clean reads for N46, N47, N48, N49 and RPh, respectively. We removed the low-quality raw reads, and assembled *de novo* the remaining reads by Trinity software. We obtained 154,458 transcripts and 104,358 unigenes (Additional file 9: Table S6). Then, unigenes were searched against the NCBI non-redundant (NR) protein database using the BLASTALL package and further aligned to the databases such as GO, KEGG and KOG using BLASTX for the functional annotations.

### Differential expression analysis of miRNAs

**Figure 1:**
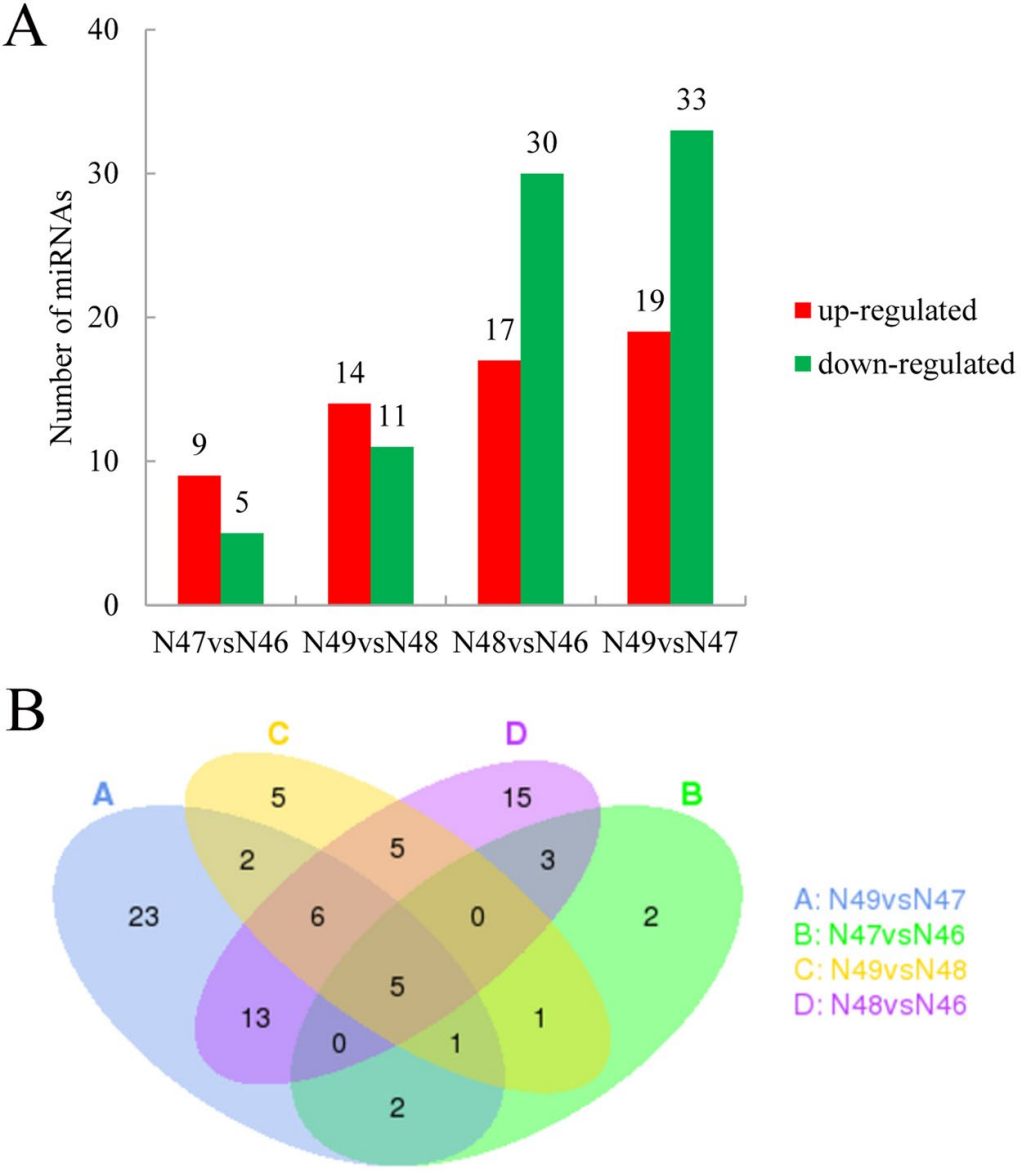
The expression of differential miRNAs in *M*. *albus*. (**A**) The number of up- and down-regulated genes in comparisons of N47 vs N46, N49 vs N48, N48 vs N46 and N49 vs N47. (**B**) The Venn diagrams of differential expression miRNAs from N47 vs N46, N49 vs N48, N48 vs N46 and N49 vs N47.

The differential miRNAs were significantly expressed with more than one log_2_ fold change and qvalue lower than 0.01 which were in different levels and comparison groups. In different coumarin expressions with a low β-glucosidase activity level between N48 and N46, 47 miRNAs were differentially expressed in these two genotypes. Among these differentially expressed miRNAs, 17 miRNAs were up-regulated and 30 miRNAs were down-regulated. A total of 52 differentially expressed miRNAs were identified between N49 and N47 in a high level of β-glucosidase activity. Of these miRNAs, 19 were up-regulated and 33 were down-regulated. Fourteen miRNAs (5 up-regulated and 9 down-regulated) and 25 miRNAs (11 up-regulated and 14 down-regulated) were significantly differentially expressed between N47 and N46 and between N49 and N48, respectively (Fig. 1A). We also found that 24 miRNAs overlapped between N48 vs N46 and N49 vs N47 (Fig. 1B). More detailed information on the different miRNAs is shown in Additional file 10: Table S7. We found that *M*. *albus* represented the same coumarin level with a similar miRNAs expression pattern, demonstrating that genotypes with the same coumarin level clustered together according to their miRNA expression (Additional file 11: Fig. S4).

**Figure 2:**
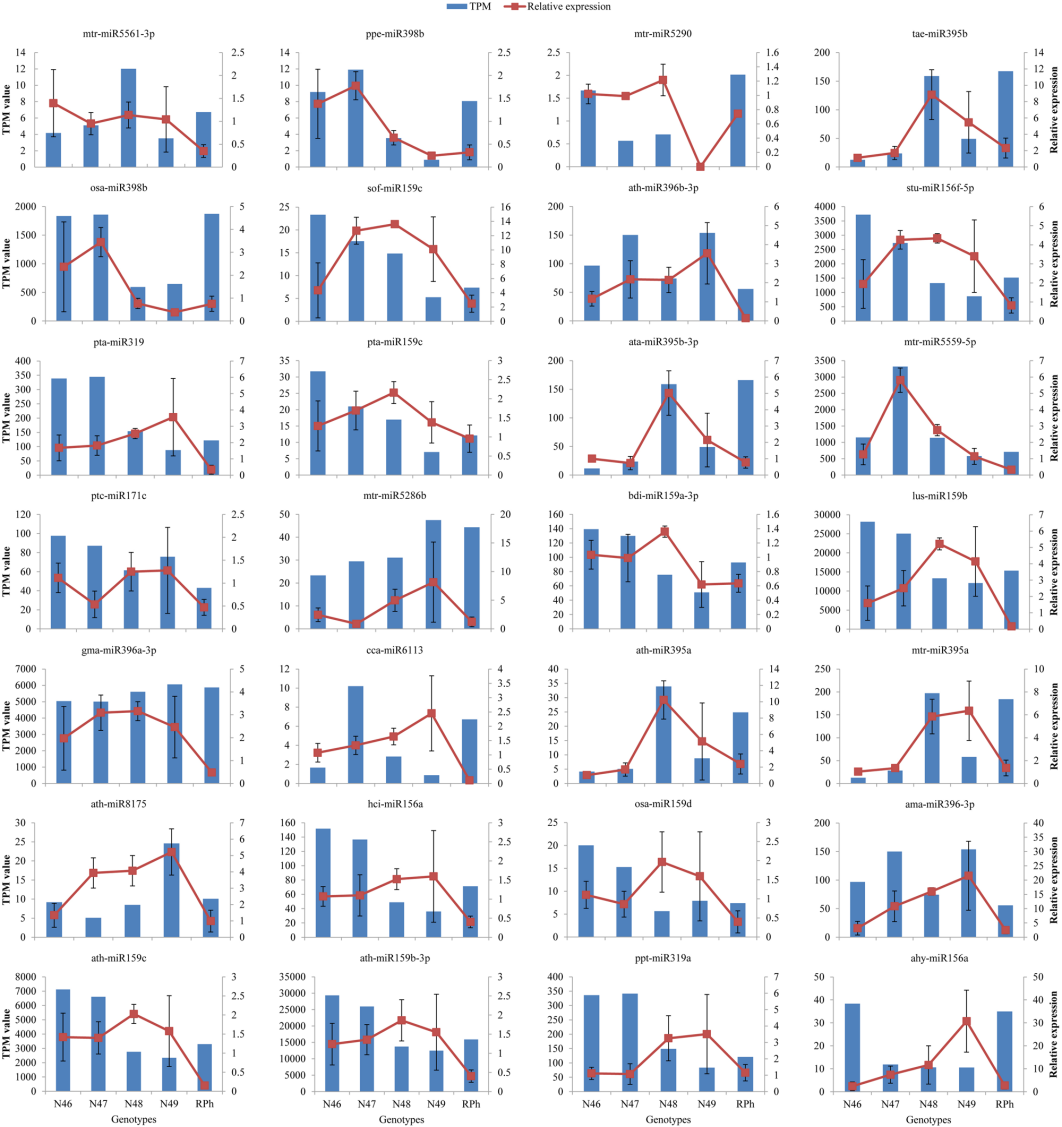
Expression profiles of miRNAs in the five different genotypes of *M*. *albus*. Validation of the expression of 28 miRNAs using qRT-PCR. The blue bar graph indicates the small RNA sequencing results, and red line graph represents the qRT-PCR results. Data are mean ± SE from three biological replicates.

To identify the results of the miRNA sequencing and bioinformatics analysis, totally 28 known miRNAs with different expression profiles were selected randomly, and their expression profiles were used to verify the sequencing results by quantitative real-time polymerase chain reaction (qRT-PCR) analysis. The qRT-PCR expression pattern and TPM value of these miRNAs are shown in Fig. 2 and Table S8. The results showed that the majority of relative expression obtained by qRT-PCR was consistent with the sequencing results because the expression trends are similar. This indicated that the miRNA sequencing data here were reliable. For example, ppe-miR398b, mtr-miR5290, tae-miR395b, osa-miR398b, ath-miR396b-3p, mtr-miR5559-5p, ath-miR395a and mtr-miR395a showed a similar expression profile between sequencing and qRT-PCR. However, some miRNAs showed that the qRT-PCR results were inconsistent with the sequencing results, i.e., stu-miR156f-5p, pta-miR319, ath-miR159c, ppt-miR319a, ahy-miR156a, and lus-miR159b.

### Prediction and annotation of miRNA target genes

In order to gain insight into the functions of the identified *M*. *albus* miRNAs, the target genes of these miRNAs were predicted using the psRobot_tar program. The 114 known miRNA families had 4155 affiliated target genes and 2 of 76 novel miRNAs had 14 target genes. Moreover, some miRNAs targeted a single gene, whereas the other miRNAs targeted multiple genes. To better investigate the functions of miRNAs, the target genes were analysed with functional analysis, GO annotations and the KEGG pathway.

**Figure 3:**
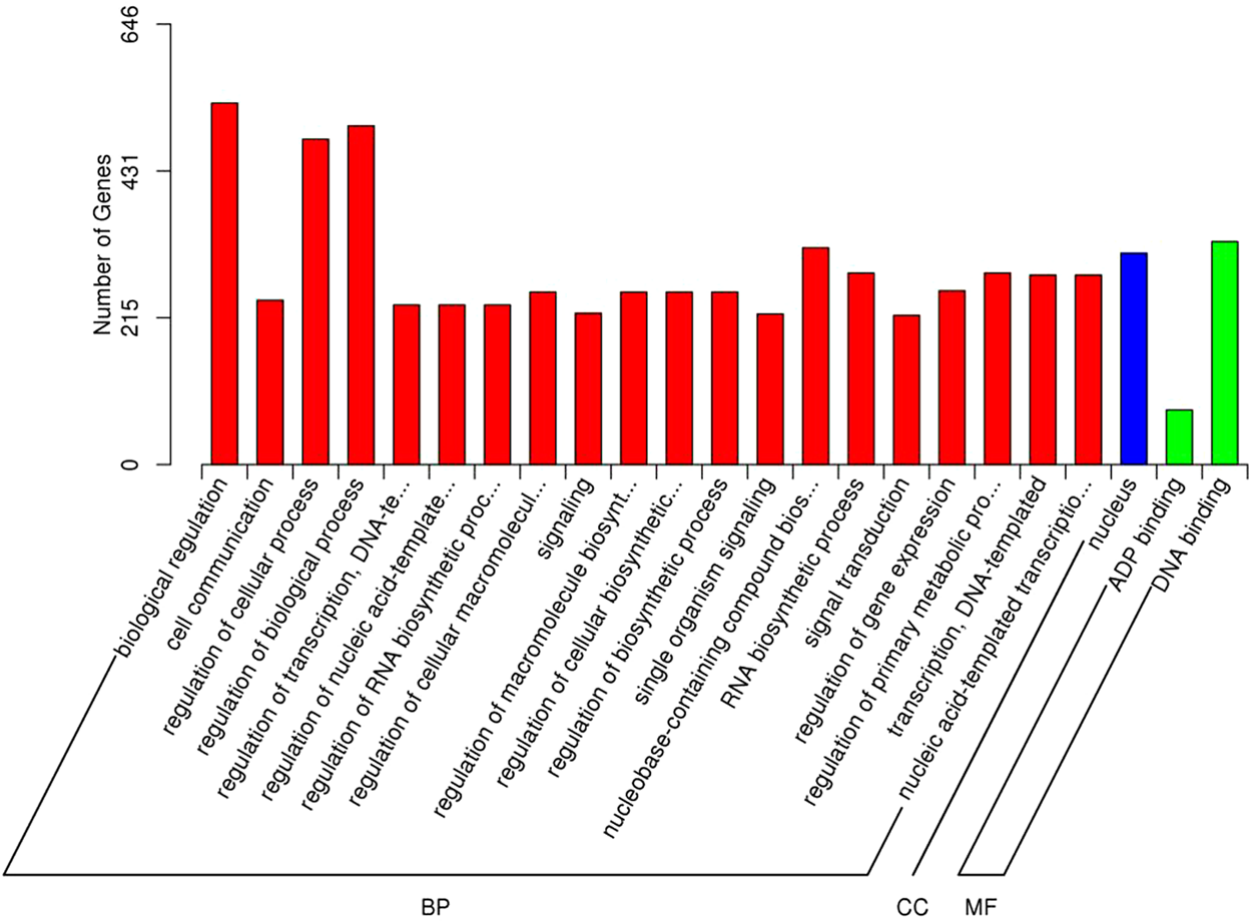
Gene ontology (GO) classification of miRNAs target genes. The results are summarized under three main GO categories: BP-biological process, CC-cellular component and MF-molecular function.

**Figure 4:**
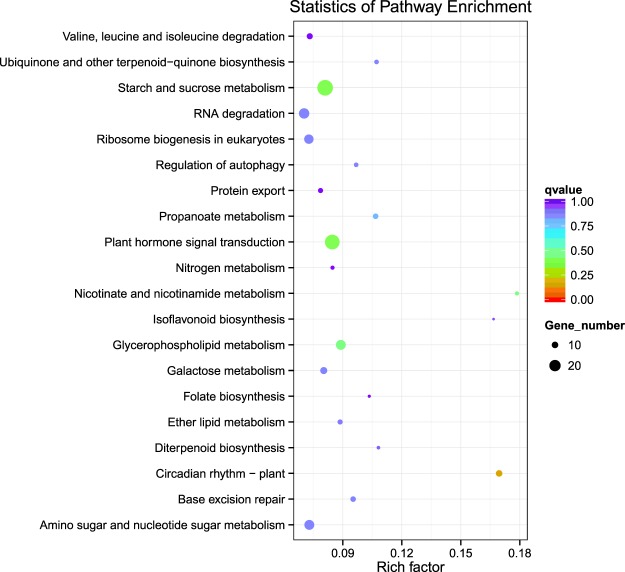
Top of 20 pathways assignment based on Kyoto Encyclopedia of Genes and Genomes (KEGG) database.

The results of GO analysis demonstrated that the target genes of the miRNAs could be enriched into 3128 GO terms (Additional file 12: Table S9). Among them, 20 biological process categories, 2 cell components categories and 1 molecular function category were significantly enriched (Fig. 3). Under the biological process, biological regulation, regulation of biological process, regulation of cellular process and others were significantly enriched. For the cellular component category, the nucleus was the most highly represented group. In the molecular function, ATP binding and DNA binding were significantly enriched. From the KEGG analysis, one hundred and seven pathways were found (Additional file 13: Table S10), and the top 20 enrichment pathways are shown in Fig. 4. Circadian rhythm, plant hormone signal transduction, starch and sucrose metabolism, isoflavonoid biosynthesis and nicotinate and nicotinamide metabolism were the most frequently represented pathways.

Furthermore, qRT-PCR was performed on 32 differentially expressed target genes randomly selected from the expression profile data to validate the assembly and annotation of the RNA-Seq data. The qRT-PCR expression patterns and FRKM values of these target genes are shown in Additional file 14: Fig. S5. The results showed that the majority of the expression levels of these selected genes obtained by qRT-PCR were consistent with the sequencing results.

### Correlation analysis between miRNAs and target genes

**Figure 5:**
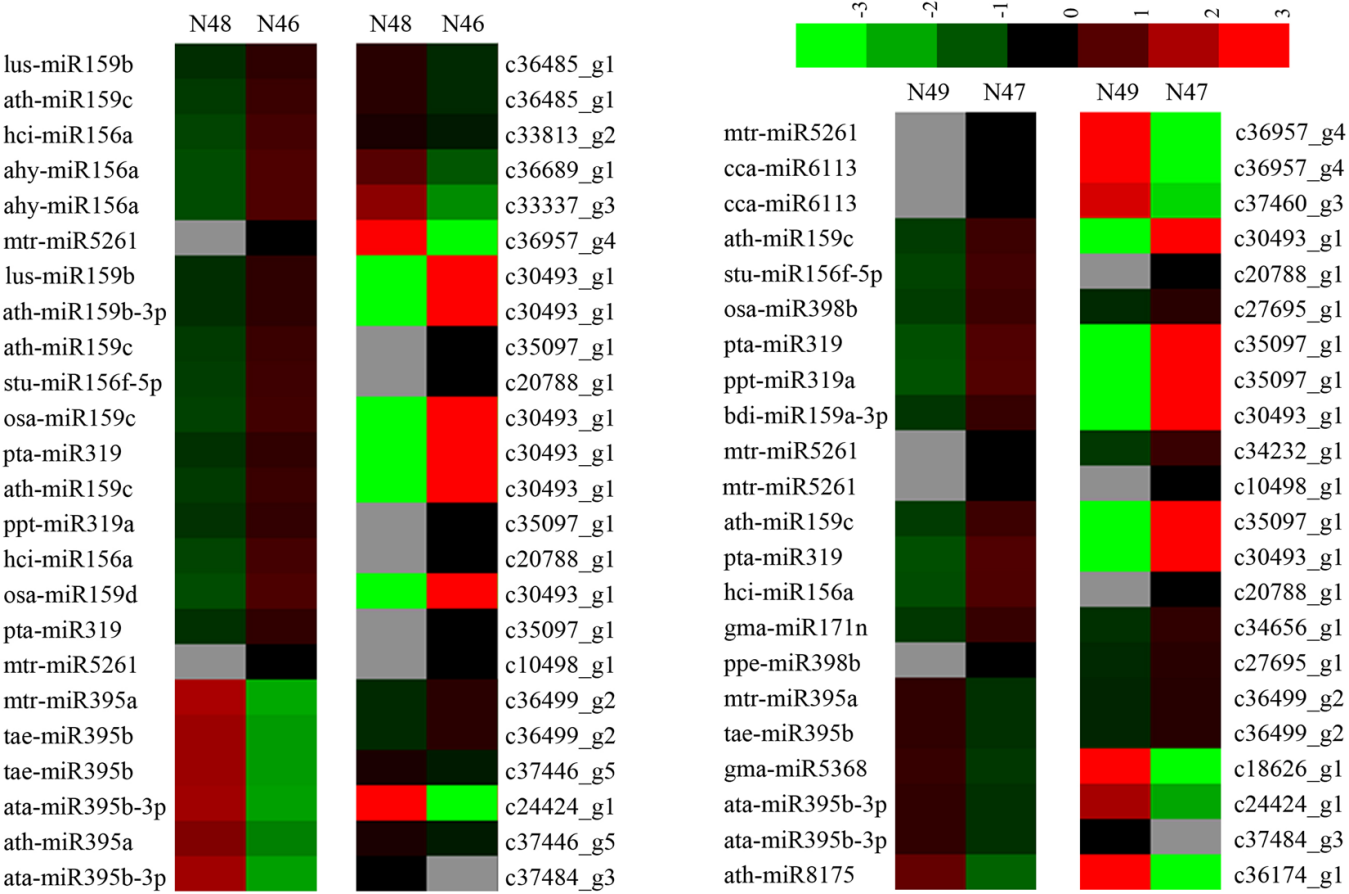
A combined view of correlation expressions between miRNA and its target gene compared in N48 vs N46 and N49 vs N47. The left side of heat map show miRNA expression level, and the right side show corresponding target gene expression levels of both N48 vs N46 and N49 vs N47. Up and down regulation in the expression were based on normalize data (color bar at the top) generated by Cluster 3.0 software.

**Figure 6:**
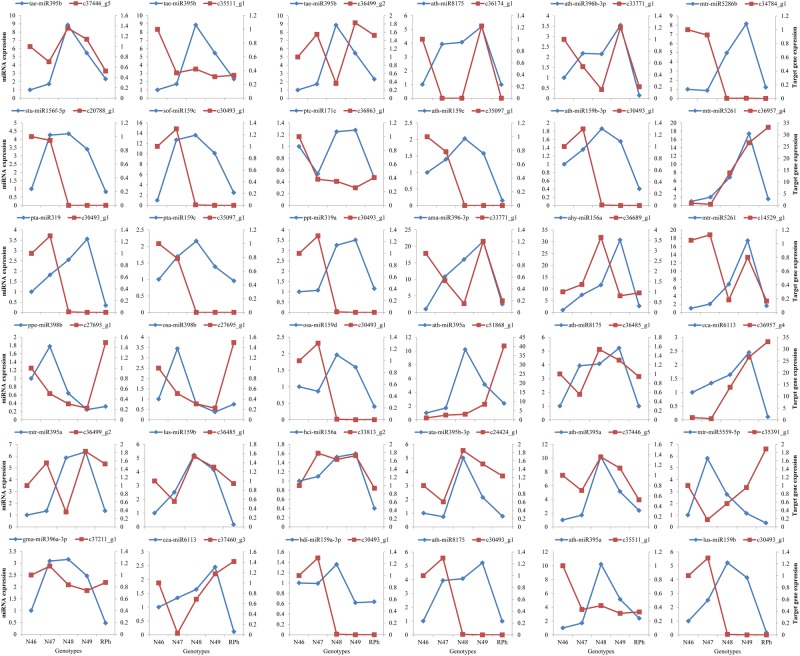
Validation of the expression of miRNAs and their target genes using qRT-PCR in five genotypes of *M*. *albus*. The blue lines indicate the miRNAs relative expression, and red represents the target genes relative expression. The relative expression was calculated using 2^−∆∆CT^ method.

To better understand the possible roles of the miRNAs and their target genes, we analyzed the relationship between differentially expressed miRNAs and the expression of their target genes differentially combined with the results of the transcriptome sequencing. It was observed that 53 miRNA-target pairs showed correlations which contained 35 miRNAs and 30 target genes (Table 3). And these miRNA-target pairs had a number of functions, such as multidrug resistance protein, regulation of transcription, photosystem II stability, assembly factor, DNA binding, high-affinity nitrate transporter and other proteins were involved in various biological processes from the functional annotations. In a different comparison group, various numbers of correlated miRNA-target pairs were found. In the N48 vs N46 group, 8 miRNA-target pairs showed a negative regulation pattern of 24 miRNA-target pairs. Meanwhile, 22 miRNA-target pairs had the correlation expression while 5 miRNA-target pairs were negative expressed between N49 and N47 (Fig. 5 and Table S11). We further validated the expression of the identified miRNAs and investigated the expression of their target genes using qRT-PCR. As shown in Fig. 6, 36 miRNAs and their targets were randomly selected to analyse the expression patterns in *M*. *albus*. Comparing N48 and N46, for example, mtr-miR395a and tae-miR395b with their target gene c36499_g2 presented negative correlation, whereas ath-miR395a and tae-miR395b with their target gene c37446_g5 showed a positive expression. These results are similar to the expression pattern in Fig. 5.

**Table 3:** Differentially expressed miRNAs and differentially expressed target genes identified in *M*. *albus*.

miRNA	miRNA sequence	Target gene	Target gene annotation
ahy-miR156a	TGACAGAAGAGAGAGAGCAC	c30829_g1	REV protein (anti-repression trans-activator protein)
		c33337_g3	Choline/ethanolamine kinase
		c36689_g1	—
aly-miR172e-3p	GAATCTTGATGATGCTGCAT	c35325_g2	Serine carboxypeptidase-like clade IV
		c36434_g1	Phosphomethylpyrimidine synthase
ama-miR396-3p	AAGCTCAAGAAAGCTGTGGGA	c33771_g1	CXCR4 Chemokine receptor N terminal
ata-miR395b-3p	AAGTGTTTGGGGGAACTC	c24424_g1	GTP cyclohydrolase I
		c37484_g3	Kinesin-like calmodulin binding protein
ath-miR159b-3p	TTTGGATTGAAGGGAGCTCTT	c30493_g1	Transposon Ty3-I Gag-Pol polyprotein
ath-miR159c	TTTGGATTGAAGGGAGCTCCT	c30493_g1	Transposon Ty3-I Gag-Pol polyprotein
		c35097_g1	Homeobox associated leucine zipper, regulation of transcription, DNA-templated, DNA binding
		c36485_g1	Multidrug resistance protein, MATE family
ath-miR171b-3p	TTGAGCCGTGCCAATATCACG	c34656_g1	Zinc finger CCCH domain-containing protein
ath-miR172e-3p	GGAATCTTGATGATGCTGCAT	c35325_g2	Serine carboxypeptidase-like clade IV
		c36434_g1	Phosphomethylpyrimidine synthase
ath-miR395a	CTGAAGTGTTTGGGGGAACTC	c35511_g1	Embryo defective 3006 protein, putative
		c37446_g5	Photosystem II stability/assembly factor HCF136
		c51868_g1	ER vesicle integral membrane protein involved in establishing cell polarity, signaling and protein degradation
ath-miR396b-3p	GCTCAAGAAAGCTGTGGGAAA	c33771_g1	CXCR4 Chemokine receptor N terminal
ath-miR8175	GATCCCCGGCAACGGCGCCA	c36174_g1	FOG: Transposon-encoded proteins with TYA, reverse transcriptase, integrase domains in various combinations
bdi-miR159a-3p	CTTGGATTGAAGGGAGCTCT	c30493_g1	Transposon Ty3-I Gag-Pol polyprotein
cca-miR6113	TCTGAAACTCAAGAACACGTTG	c36957_g4	Resistance protein
		c37460_g3	TIR-NBS-LRR RCT1 resistance protein
gma-miR171n	TTGAGCCGCGTCAATATCTTA	c34656_g1	Zinc finger CCCH domain-containing protein
gma-miR5368	GGACAGTCTCAGGTAGACA	c18626_g1	Predicted oxidoreductase
hci-miR156a	TGACAGAAGAGAGTGAGTAC	c20788_g1	—
		c33813_g2	Adenylate kinase
lus-miR159b	TTTGGATTGAAGGGAGCTCTC	c30493_g1	Transposon Ty3-I Gag-Pol polyprotein
		c36485_g1	Multidrug resistance protein, MATE family
mdm-miR159a	CTTGGATTGAAGGGAGCTCC	c30493_g1	Transposon Ty3-I Gag-Pol polyprotein
mtr-miR169h	TGAGCCAAAGATGACTTGCCGG	c33282_g1	Plant OB fold protein, putative
mtr-miR395a	ATGAAGTGTTTGGGGGAACTC	c36499_g2	GD3A, dentin sialophosphoprotein, putative
mtr-miR5261	TCATTGTAGATGGCTTTGGCT	c10498_g1	TMV resistance protein N
		c14529_g1	Hypothetical protein, glycosyl hydrolase family 43 protein
		c34232_g1	High-affinity nitrate transporter
		c36957_g4	Resistance protein
mtr-miR5286b	ACAAACTGGAGGCAAGGGACAGGA	c34784_g1	MATE efflux family protein
mtr-miR5559-5p	TACTTGGTGAATTGTTGGATC	c35391_g1	Sieve element-occluding protein
osa-miR159c	ATTGGATTGAAGGGAGCTCCA	c30493_g1	Transposon Ty3-I Gag-Pol polyprotein
osa-miR159d	ATTGGATTGAAGGGAGCTCCG		
osa-miR398b	TGTGTTCTCAGGTCGCCCCTG	c27695_g1	hypothetical protein
ppe-miR398b	CGTGTTCTCAGGTCGCCCCTG	c27695_g2	hypothetical protein
ppt-miR319a	CTTGGACTGAAGGGAGCTCC	c35097_g1	Homeobox associated leucine zipper, regulation of transcription, DNA-templated, DNA binding
pta-miR159a	TTGGATTGAAGGGAGCTCCA	c30493_g1	Transposon Ty3-I Gag-Pol polyprotein
pta-miR159c	CTTGGATTGAAGGGAGCTCCC	c30493_g1	
pta-miR319	TTGGACTGAAGGGAGCTCC	c30493_g1	
		c35097_g1	Homeobox associated leucine zipper, regulation of transcription, DNA-templated, DNA binding
ptc-miR171c	AGATTGAGCCGCGCCAATATC	c36863_g1	Replication factor A protein
sof-miR159c	CTTGGATTGAAGGGAGCTCCT	c30493_g1	Transposon Ty3-I Gag-Pol polyprotein
stu-miR156f-5p	CTGACAGAAGAGAGTGAGCA	c20788_g1	—
tae-miR395b	TGAAGTGTTTGGGGGAACTC	c35511_g1	Embryo defective 3006 protein, putative
		c36499_g2	GD3A, dentin sialophosphoprotein, putative
		c37446_g5	Photosystem II stability/assembly factor HCF136

### Identification of target genes involved in the coumarin pathway

**Figure 7:**
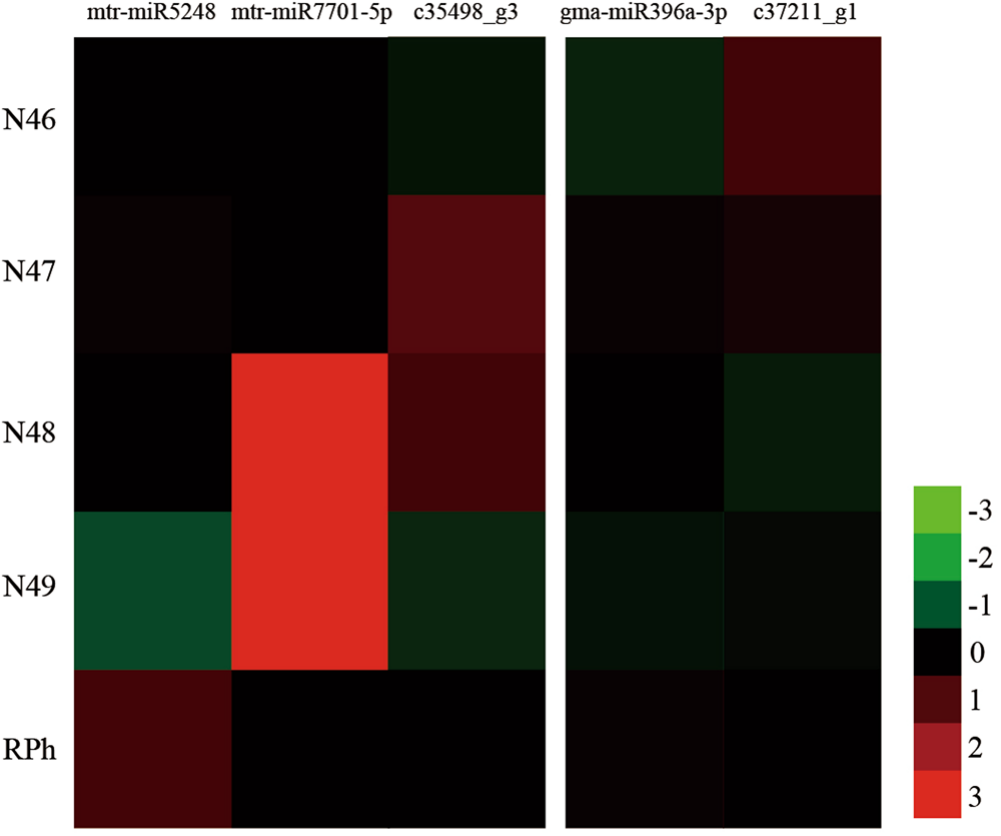
The expression of three miRNAs and their target HCT genes in five genotypes of *M*. *albus*. The red color represents high expression levels, and the green color represents low level expression.

From the functional annotations of 4169 miRNA target genes, two shikimate O-hydroxycinnamoyltransferase (HCT) genes were found to be associated with coumarin synthesis pathways, including gene c35498_g3 for mtr-miR5248 and mtr-miR7701-5p and gene c37211_g1 for gma-miR396a-3p. Largely varied expression was observed for these miRNAs and their target genes in the five genotypes (Fig. 7). As an example, mtr-miR7701-5p showed higher expression in N48 and N49 than in N46 and N47. Additionally, miR7701-5p with its target gene c35498_g3 presented a positive expression in N48, whereas in N49 it showed the negative expression.

## Discussion and Conclusion

miRNAs play important roles in the regulation of plant growth and development, biotic and abiotic stress responses and other biological processes. Various miRNAs in different plants have been discovered and characterized, and revealed the molecular mechanisms in regulating miRNAs^20^. Although miRNAs have been reported in many plants, such as *Arabidopsis thaliana*^21^, maize^22^, wheat^23^, and other plants^24,25^, no study has been performed on *M*. *albus*, and the miRNAs and their target genes of *M*. *albus* remain unknown. In this study, we studied the regulation of *M*. *albus* miRNAs and function of their target genes using a high-throughput sequencing analysis. The accuracy of the sequencing and the expression of the miRNAs and the miRNA target genes were analysed through qRT-PCR.

The small RNA length distribution of *M*. *albus* indicated that the 24 nt small RNAs were the highest abundance, representing 44.26%, 44.36%, 40.78%, 42.95% and 43.19% in the five genotypes (Additional file 3: Fig. S1). Similar results have been found in the previous studies with other plant species, such as *Medicago sativa* L.^26^, *Vigna mungo*^27^ and foxtail millet^28^. Therefore, as in other plants, the 24 nt small RNAs may also be involved in critical functions in *M*. *albus*. Small RNAs can be annotated into different categories, including rRNA, tRNA, snRNA, snoRNA, repeat associated sRNA, TAS, and sRNAs that could not be annotated. In previous studies, the majority of them were mapped to other unannotated and not annotated RNAs in the sRNA libraries^29,30^, and the results of our present study were similar with very few known and novel miRNAs. This suggests that many miRNAs currently have not been recognized. The expression level of various miRNAs was significantly different which had been reported previously^31,32^. Similarly to the previous study, the expression level of identified 417 known miRNAs and 76 novel miRNAs also showed a large variation in our current study. In this study, the number of miRNA family members varied greatly, ranging from 1 to 35 (miR156), similar to the previous studies^14,33^. In addition, most of the predicted target genes annotated in consistent with the biological functions of the genes in foxtail millet^33^, *Arabidopsis thaliana*^34^, and wheat^35^. In short, our results suggests that the high-throughput sequencing analysis for *M*. *albus* miRNAs and their target genes was reliable mainly due to the study for the length distribution of small RNA, the function, expression and regulation of miRNAs and their target genes in *M*. *albus* were consistent with previous researches.

Furthermore, to gain insight into the regulatory function of miRNAs, a large number (4169) of target genes were identified and annotated by mapping to the GO and KEGG databases. In this study, the prediction of the target gene showed that miRNAs can target genes related to plant growth and development, including circadian rhythm, starch and sucrose metabolism, and plant hormone signal transduction. Previous studies had shown that miRNA families in different plants perform analogous regulatory functions^36^. More study of the miRNAs and their target genes will help further understanding the regulatory mechanism and function of *M*. *albus* miRNAs. The incomplete mRNA database may limit the comprehensive identification of target genes as well. Consequently, further construction of small RNA and libraries from different tissues and developmental stages should provide more insight into the network between miRNAs and their target genes in *M*. *albus*.

Investigations of coumarin biosynthesis were conducted during the 1960s and 1970s with the help of tracer-feeding experiments^37^. Several enzymes related to this biosynthesis have been identified, such as trans-cinnamate, trans-2-coumarate, trans-2-coumarate-β-D-glucoside and cis-2-coumarate^38^. However, to date, there is a general lack of gene information for the enzymes involved in the coumarin biosynthesis pathway. Recently, several branch pathways and enzymes catalysing coumarin-formation reactions in other plant species have been identified with the help of modern synthesis and molecular techniques^39–41^. Phenylalanine ammonia-lyase (PAL), which converts phenylalanine to cinnamic acid, is the first enzyme in the coumarin biosynthesis pathway. Trans-cinnamate 4-monooxygenase (C4H) then adds a hydroxyl group to produce 4-coumarate acid, and CoA is linked by 4-coumarate-CoA ligase (4CL) ^42^. HCT belongs to the large family of BAHD-like acyltransferases^43^, a key enzyme in the phenylpropanoid and lignin biosynthesis pathway. A study on Arabidopsis demonstrated that HCT gene silencing led to significant changes in lignin content^44^. The role of HCT in coumarin biosynthesis also has been reported^3^. In our study, two HCT genes were identified, and they showed differential expression in five different genotypes. The regulation of coumarin biosynthesis is complex. Major details remain unresolved, and many of the P450-dependent enzymatic steps are largely unknown. Considering the importance of coumarin, the functions of the miRNA and their target genes which will be involved in the coumarin biosynthesis pathway need further investigation. It is important to identify *Melilotus* species and genotypes with low coumarin levels.

More positive and mixed correlations were found in our analysis of the expression pattern of the selected miRNA-target pairs than we had expected, which was similar to previous studies on Chinese cabbage^45^ and *Salix matsudana*^46^. Such a comparison of the different coumarin contents in *M*. *albus* to identify the differentially expressed miRNAs may lead to further understanding of the post-transcriptional regulation of coumarin biosynthesis and its regulatory network. Currently, the differentially expressed miRNAs regulated from different comparisons among five *M*. *albus* genotypes have not been implicated in the regulation of coumarin biosynthesis. For example, we compared the expression levels of miRNAs between N48 and N46 (different coumarin expression at a low β-glucosidase activity level) and classified a total of 15 differentially expressed miRNAs with down- and up-regulation in response to target genes. MiR156, miR159, miR319 and miR5261were found to be significantly abundant in N46. Thus, these miRNAs may play important roles in coumarin biosynthesis.

In conclusion, this is the first time high-throughput sequencing has been employed to identify miRNAs and their target genes from five *M*. *albus* NILs. We identified 417 known and 76 novel miRNAs in five genotypes of *M*. *albus*. The predicted 4196 target genes were performed the functions annotation using GO and KEGG. Additionally, two target HCT genes were computationally predicted to involve in the coumarin biosynthesis pathway with three miRNAs. This study will provide useful information for future research on the functions and molecular regulatory mechanisms of miRNA and their target genes in *M*. *albus*. However, the predicted miRNA and their targets need to be further evaluated with its reference genome in case of any false-positive predictions. Furthermore, these selected miRNAs should be functional identified to understand the regulatory roles in *M*. *albus* coumarin biosynthesis pathway.

## Methods

### Plant materials and coumarin content determination

The five genotypes of *M*. *albus*, near-isogenic lines, N46, N47, N48, N49 and the recurrent male parent of RPh, were obtained from an initial cross of cucubb biennial plants × CuCuBB plants of RPh. Here, Cu/cu and B/b are two pairs of alleles affecting coumarin content and β-glucosidase activity, respectively. Then, cucubb segregates, which differ in coumarin content and β-glucosidase activity, were underwent six successive backcrosses to the RPh^47^. The seeds of five genotypes were planted in 20 cm plots containing agricultural soil with a photoperiod of 16 h light at 26 °C and 8 h dark at 18 °C in a greenhouse at Lanzhou University, Gansu Province, China. The leaves (three individuals from each genotype plant) were collected at the flowering stage. These young leaves were immediately frozen in liquid nitrogen and then stored at −80 °C for further use.

Fresh leaves from three individuals of each genotype were collected for the determination of coumarin content. For the assay of coumarin content, each replicate of five genotypes was combined and ground in a mill to pass a 1 mm screen for coumarin determination. 0.1 g of ground material was extracted twice with 1 mL of 60% ethanol at 30 °C for 30 min. Coumarin was quantified by high-performance liquid chromatography (HPLC) using the mobile phase of methanol-water (65:35) through an Agilent-XDB C18 column^48^. For the measurement of β-glucosidase activity, an enzyme-linked immunoassay assay (ELISA) was performed with a β-glucosidase activity assay kit (MeilianBio Co., Ltd, Shanghai, China) following the manufacturer’s instructions. As shown in Table 4, the coumarin content in N48 and N49 was significantly higher (*p* < 0.05) than that in N46 and N47. For the same level of coumarin, N47 and N49 had higher β-glucosidase activity than N46 and N48, respectively. RPh had a similar coumarin content and β-glucosidase activity to N49.

**Table 4:** The information of coumarin content and β-glucosidase activity in five genotypes of *M*. *albus*.

Line	Genotype	Coumarin (%)	β-glucosidase (mU/L)
N46	cucubb	0.25 ± 0.07b	0.64 ± 0.13c
N47	cucuBB	0.27 ± 0.11b	2.02 ± 0.14b
N48	CuCubb	1.37 ± 0.14a	0.54 ± 0.09c
N49	CuCuBB	1.10 ± 0.21a	2.20 ± 0.05b
RPh	CuCuBB	1.22 ± 0.08a	2.59 ± 0.10a

### RNA isolation, small RNA and mRNA library construction and sequencing

Total RNA of six fresh leaves as a sample in each *M*. *albus* genotype was isolated using the RNAprep pure Plant RNA Purification Kit (Tiangen Biotech, Beijing, China). Each sample was constructed three libraries. RNA degradation and contamination was monitored on 1% agarose gels. And the total RNA quantity and purity were determined with the OD260/280 ratio and checked using the NanoPhotometer^®^ spectrophotometer (IMPLEN, CA, USA). RNA integrity was assessed using the RNA Nano 6000 Assay Kit of the Agilent Bioanalyzer 2100 system (Agilent Technologies, CA, USA). Small RNA and mRNA libraries were prepared using NEBNext^®^ Multiplex Small RNA Library Prep Set for Illumina^®^ (NEB, USA.) and NEBNext^®^ Ultra™ RNA Library Prep Kit for Illumina^®^ (NEB, USA) following the manufacturer’s recommendations, respectively, and index codes were added to attribute sequences to each sample. Then we performed single-end reads and paired-end reads on the Illumina Hiseq. 2500 platform from small RNA and mRNA libraries, respectively.

### Identification of known and novel miRNA

After removing reads containing ploy-N, with 5′ adapter contaminants, without 3′ adapter or the insert tag, containing ploy A or T or G or C and low quality reads from raw data, the small RNA tags ranged from 18–30 nt were mapped to the reference sequence by Bowtie^49^ with no mismatches using miRBase 20.0 as a reference. The known miRNA and the secondary structures were obtained through the software mirdeep2^50^ and srna-tools-cli. The novel miRNA prediction was used the software miREvo^51^ and mirdeep2^50^ with the characteristics of hairpin structure of miRNA precursor. The parameter used to screen for “novel” miRNAs predicted using miRDeep2 were as follows: (a) Delete miRDeep2 score: <100; (b) The ratio of mature miRNA vs. miRNA*; and (c) we screened the predicted miRNAs strictly according to the hairpin structure, with only a 2-nt overhang, which is the hallmark of a bona fide miRNA. And the secondary structure, the Dicer cleavage site and the minimum free energy of the small RNAs were explored the prediction of novel miRNA. The sRNAs can be annotated into different categories, including rRNA, tRNA, snRNA, snoRNA, repeat associated sRNA, TAS, and sRNAs that could not be annotated.

### Differential expression of miRNAs

The differential expression miRNAs analysis among the five libraries was performed using the DEGseq^52,53^ R package. The P-value was adjusted to get qvalue^54^. miRNA with qvalue <0.01 and |log_2_(foldchange)|> 1 were considered as the significantly differentially expressed miRNAs.

### Prediction of potential miRNA target genes

The psRobot_tar in psRobot^55^ was used for predicting the target gene of miRNA, and the same rules as previously reported^56,57^. The parameters to adjust include the following: (1) penalty score for the alignment between smRNAs and targets, which is defined by the formulas below; (2) the boundaries of essential sequence region, within which mismatches or gaps will receive double penalty scores than other regions; (3) the threshold for the total number of gaps within the smRNA and target alignment region; and (4) the region within which gaps are permitted. Degradome sequences mapped within the target sites will be analyzed and presented. To annotate the functions and pathways of the predicted target genes, the target genes of miRNAs were assigned to various GO based Wallenius non-central hyper-geometric distributions^58^ and to the KEGG which used KOBAS^59^ software to test the statistical enrichment.

### Quantitative real-time PCR analysis

To analyse the miRNA expression pattern and the correlation between miRNAs and their target genes, miRNAs and their target genes with different expression patterns were selected for qRT-PCR. cDNA was synthesized from total RNA using the miRcute miRNA First-Strand cDNA Synthesis kit (Sangon, Shanghai, China) and Mir-X™ miRNA qRT-PCR SYBR^®^ Kit (Takara, Dalian, China), according to the manufacturer’s instructions. All qRT-PCR reactions were performed with three biological and three technical replicates for each sample in 96-well plates on an ABI 7500 Real-Time PCR System. The reactions were performed in a volume of 10 μL containing 1 μL of cDNA, 5 μL of SYBR Green PCR Master Mix (Applied Biosystems), 0.5 μL of each primer and 3 μL of double-distilled water. The primers used to amplify the miRNAs and target genes are listed in Additional file 15: Table S12. The following reaction conditions were used: denaturation for 10 min at 95 °C, 40 cycles of 95 °C for 15 s, and finally 60 °C for 1 min. A melting curve analysis with 95 °C for 15 s, 60 °C for 1 min, and 95 °C for 15 s was performed to produce a dissociation curve for verification of the amplification specificity. The relative expression of the selected miRNA and target genes were normalized using U6 and β-tubulin and analysed with the 2^−∆∆Ct^ method^60^.

## Electronic supplementary material

Supplementary Information

Table S5

Table S9

Table S10

## Data Availability

The data sets supporting the results of this article are included within the article and its additional files.
