# Supplementary material for: Analysis of miRNAs and their target genes in five Melilotus albus NILs with different coumarin content
Source: Sci Rep. 2018 Sep 20;8:14138. doi: 10.1038/s41598-018-32153-3 (PMC6147922; doi:10.1038/s41598-018-32153-3)
Supplement: Supplementary file 1 — Supplementary Information [file 41598_2018_32153_MOESM1_ESM.docx]

**Analysis of miRNAs and their target genes in five *Melilotus albus* NILs with different coumarin content**

**Fan Wu^1 ¶^, Kai Luo^1 ¶^, Zhuanzhuan Yan^1^, Daiyu Zhang^1^, Qi Yan^1^, Yufei Zhang^1^, Xianfeng Yi^2 *^ and Jiyu Zhang^1 *^**

^1^ State Key Laboratory of Grassland Agro-ecosystems, Lanzhou University; Key Laboratory of Grassland Livestock Industry Innovation, Ministry of Agriculture, Lanzhou University; College of Pastoral Agriculture Science and Technology, Lanzhou University; Lanzhou, 730020, China

^2^ Guangxi Institute of Animal Sciences, Nanning, 530001, China

**^*^** Corresponding author: Xianfeng Yi and Jiyu Zhang

**^¶^** These authors contributed equally to this work.

Correspondence: [1154128631@qq.com](mailto:1154128631@qq.com); zhangjy@lzu.edu.cn


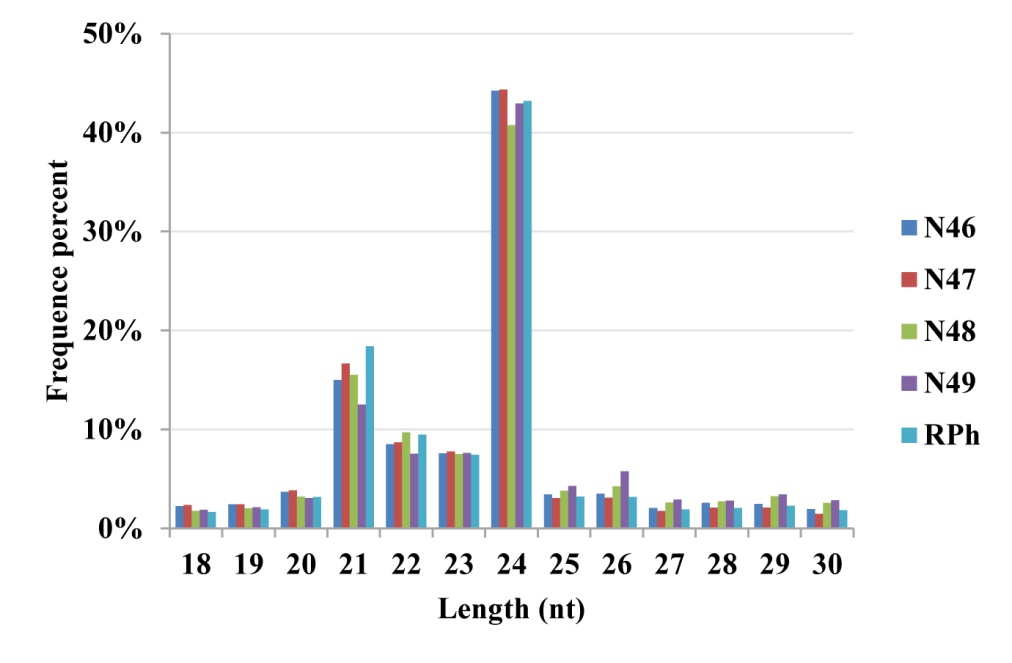


**Figure S1** **Length distributions of small RNAs identified in five genotypes of *M. albus*.** The number of sequences is expressed as a percentage of the total number of sequences in the 18-30 nt sRNA subset in each library.


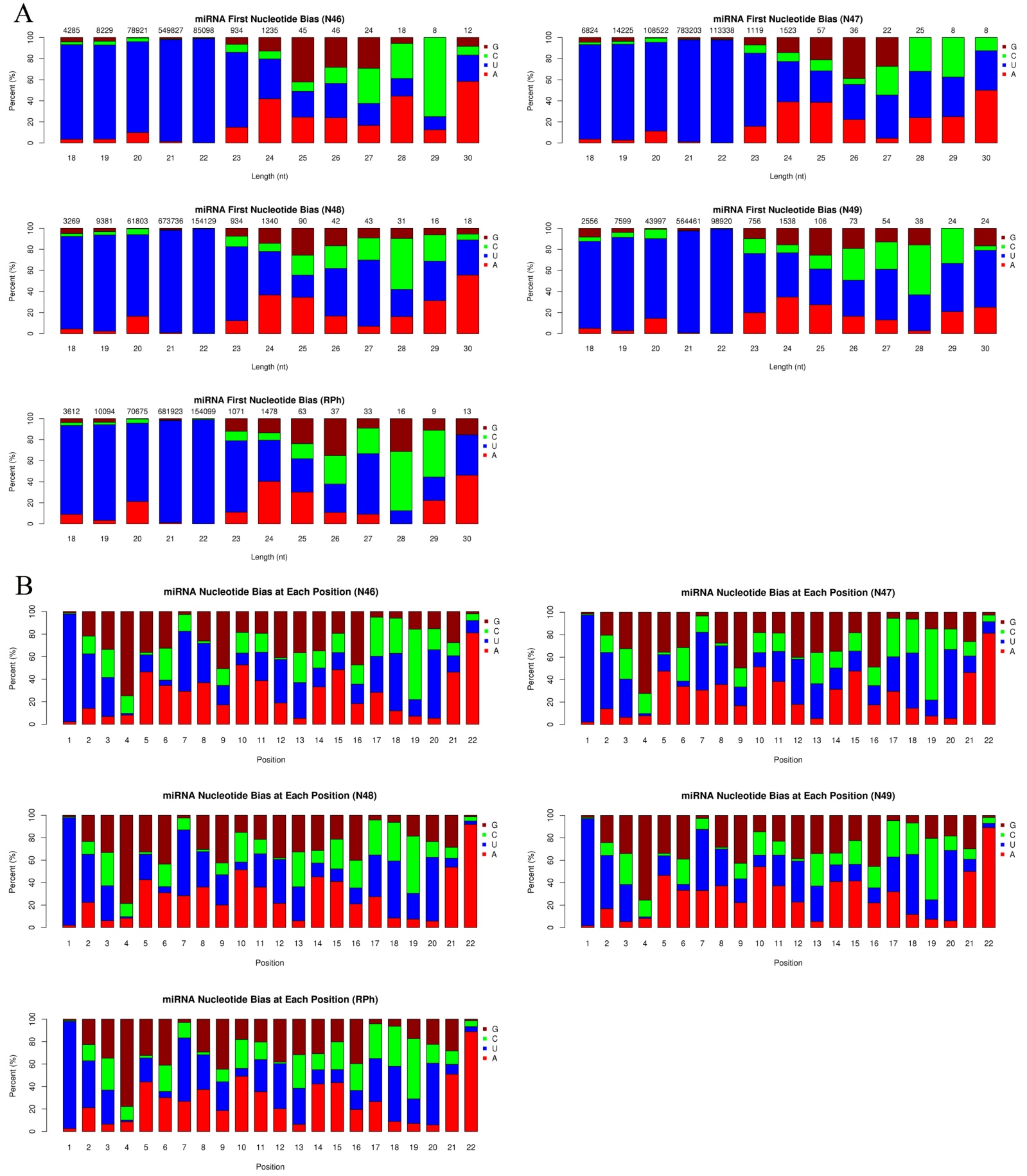


**Figure S2 Base bias of miRNAs in five genotypes of *M. albus*.** (A) First nucleotide bias for the first position of 18-26 nt miRNA; (B) miRNA nucleotide bias at each position of 22 nt miRNAs.


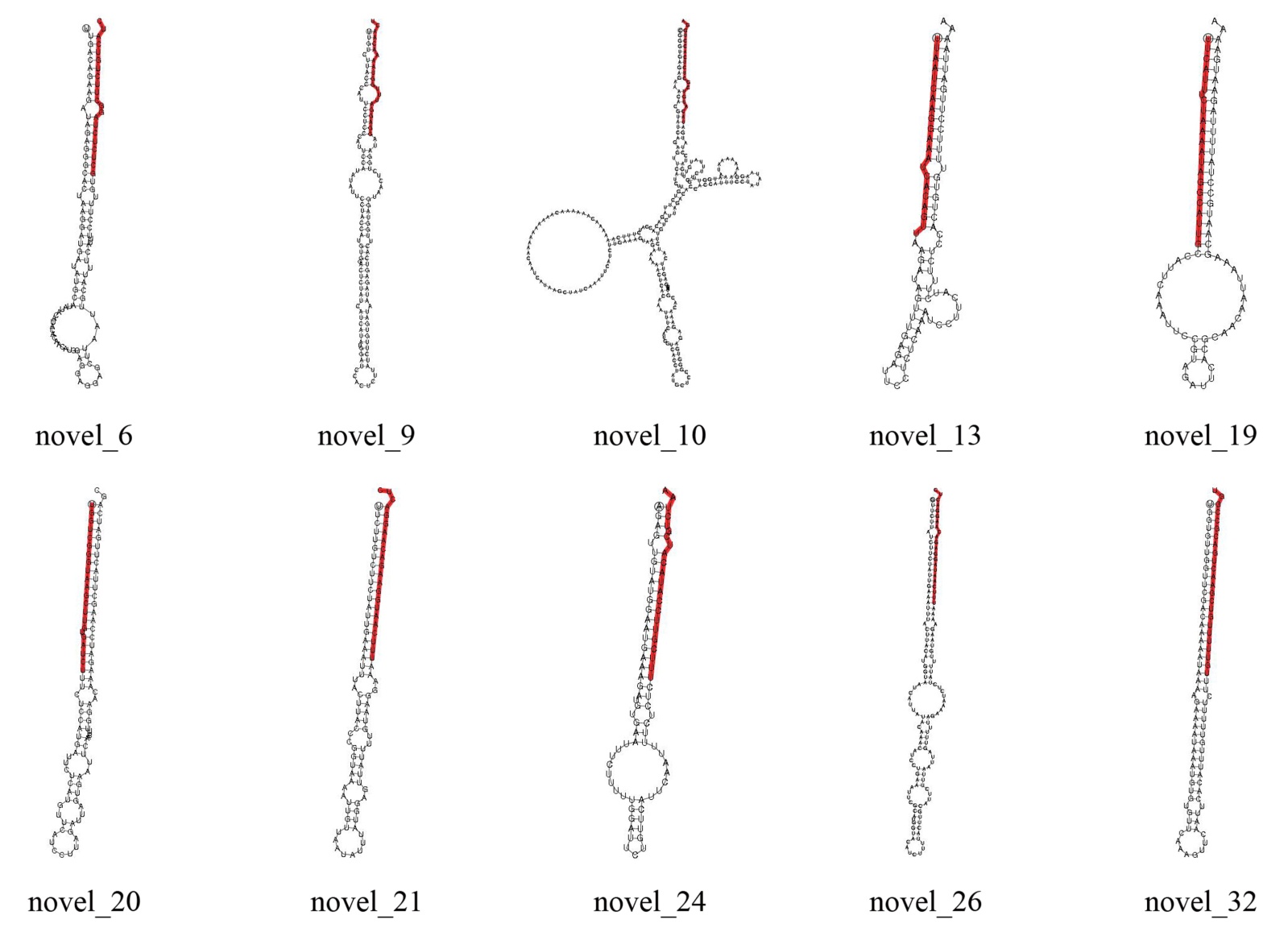


**Figure S3 Secondary structure of predicted novel miRNA precursors in *M. albus*.** Red shaded areas indicate the dominant mature miRNAs.


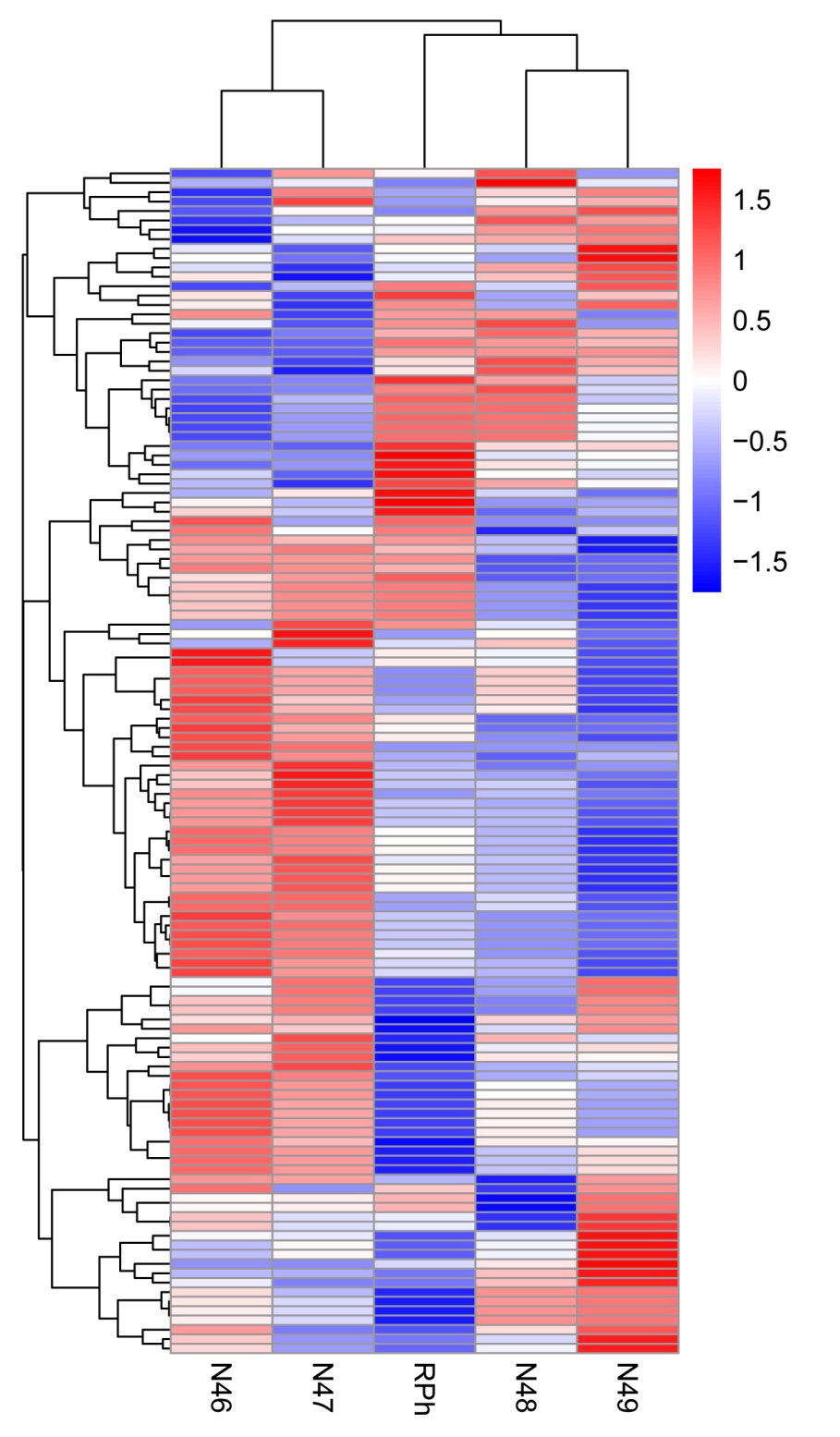


**Figure S4** **Heat-map showing expression patterns of miRNAs differentially regulation among five *M. albus* genotypes.** Each row represents a single miRNA, and each column represents a genotype library. Red indicates high abundance and blue indicates low abundance.


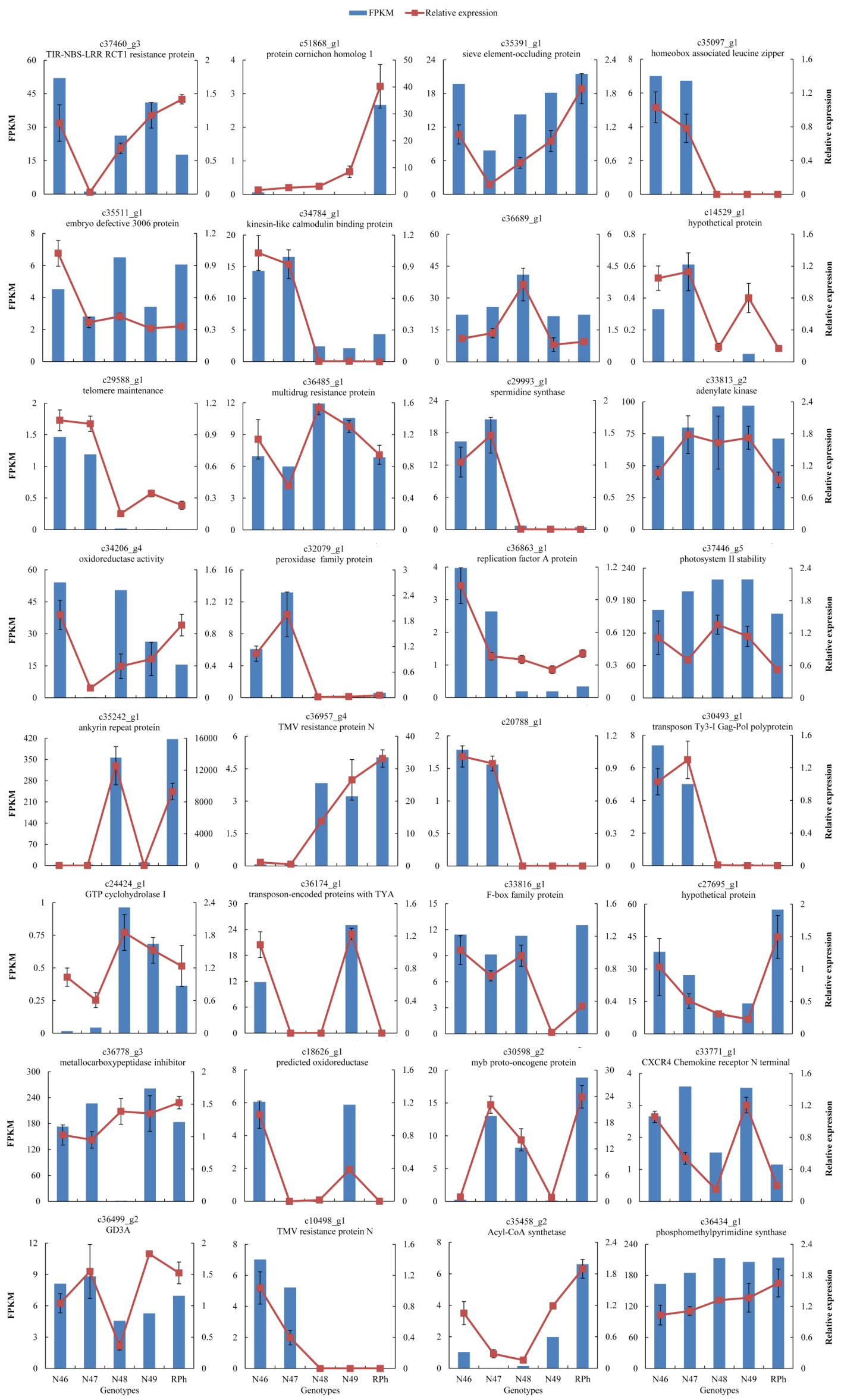


**Figure S5 Quantitative real-time PCR validate expressions of miRNAs target genes.** Left y-axis indicates genes abundance change based on FPKM values according to RNA-Seq. The relative expression was calculated using 2^-∆∆CT^ method (right y-axis). Results represent mean standard deviations (±SD) of three biological replications.

**Table S1 Statistics of transcriptome output sequencing.**

| **Genotype** | **Reads** | **Bases** | **Error rate** | **Q20** | **GC content** |
| --- | --- | --- | --- | --- | --- |
| N46 | 23828530 | 1.191G | 0.01% | 98.88% | 48.92% |
| N47 | 24763206 | 1.238G | 0.01% | 98.94% | 48.66% |
| N48 | 23510450 | 1.176G | 0.01% | 98.81% | 48.94% |
| N49 | 24496278 | 1.225G | 0.01% | 98.92% | 48.86% |
| RPh | 24240728 | 1.212G | 0.01% | 98.87% | 48.62% |

**Table S2 Summary of small RNAs sequencing reads and data cleaning.**

| **Library** | **Sample** | **total_reads** | **N% > 10%** | **low quality** | **5_adapter_contamine** | **3_adapter_null or insert_null** | **with ployA/T/G/C** | **clean reads** |
| --- | --- | --- | --- | --- | --- | --- | --- | --- |
| RRN01048 | N46 | 23828530 (100.00%) | 4796 (0.02%) | 1755 (0.01%) | 7428 (0.03%) | 423521 (1.78%) | 77533 (0.33%) | 23313497 (97.84%) |
| RRN01049 | N47 | 24763206 (100.00%) | 5081 (0.02%) | 1947 (0.01%) | 7678 (0.03%) | 451634 (1.82%) | 88928 (0.36%) | 24207938 (97.76%) |
| RRN01050 | N48 | 23510450 (100.00%) | 4515 (0.02%) | 1905 (0.01%) | 6954 (0.03%) | 351419 (1.49%) | 80079 (0.34%) | 23065578 (98.11%) |
| RRN01052 | N49 | 24496278 (100.00%) | 4834 (0.02%) | 2216 (0.01%) | 7068 (0.03%) | 511575 (2.09%) | 86446 (0.35%) | 23884139 (97.50%) |
| RRN01051 | RPh | 24240728 (100.00%) | 4804 (0.02%) | 1884 (0.01%) | 9624 (0.04%) | 466224 (1.92%) | 90296 (0.37%) | 23667896 (97.64%) |

**Table S3 The reads of the known miRNAs at the five genotypes.**

| **miRNA** | **N46** | **N47** | **N48** | **N49** | **RPh** |
| --- | --- | --- | --- | --- | --- |
| aau-miR160 | 155 | 220 | 149 | 143 | 197 |
| aau-miR168 | 1648 | 2361 | 1136 | 1254 | 1556 |
| ahy-miR156a | 46 | 21 | 15 | 12 | 52 |
| ahy-miR156b-3p | 9 | 22 | 13 | 7 | 4 |
| ahy-miR398 | 420 | 1509 | 91 | 26 | 108 |
| aly-miR156f-3p | 28 | 40 | 30 | 24 | 21 |
| aly-miR157b-3p | 9 | 22 | 13 | 7 | 4 |
| aly-miR157d-3p | 14 | 23 | 9 | 5 | 12 |
| aly-miR162a-5p | 13 | 20 | 2 | 30 | 25 |
| aly-miR165a-3p | 267 | 433 | 350 | 228 | 476 |
| aly-miR165a-5p | 0 | 1 | 0 | 0 | 1 |
| aly-miR166g-5p | 12 | 40 | 25 | 17 | 28 |
| aly-miR172e-3p | 33 | 25 | 22 | 12 | 25 |
| ama-miR396-3p | 116 | 265 | 105 | 175 | 83 |
| aqc-miR159 | 459 | 698 | 639 | 507 | 593 |
| aqc-miR171f | 352 | 398 | 253 | 148 | 143 |
| ata-miR166c-5p | 0 | 0 | 1 | 0 | 0 |
| ata-miR167f-3p | 0 | 0 | 0 | 0 | 1 |
| ata-miR393-5p | 45 | 91 | 30 | 14 | 36 |
| ata-miR395b-3p | 14 | 42 | 225 | 56 | 247 |
| ata-miR408-3p | 0 | 0 | 0 | 0 | 1 |
| ath-miR156a-5p | 14127 | 19676 | 15933 | 9911 | 14295 |
| ath-miR156g | 61 | 85 | 68 | 42 | 54 |
| ath-miR156i | 0 | 1 | 0 | 0 | 2 |
| ath-miR157a-3p | 0 | 4 | 1 | 0 | 0 |
| ath-miR157a-5p | 16 | 22 | 13 | 9 | 12 |
| ath-miR157d | 0 | 1 | 0 | 1 | 0 |
| ath-miR159a | 210090 | 298447 | 253376 | 213105 | 247328 |
| ath-miR159b-3p | 35202 | 45809 | 19457 | 14191 | 23690 |
| ath-miR159c | 8537 | 11660 | 3904 | 2662 | 4896 |
| ath-miR160a-5p | 155 | 220 | 149 | 144 | 197 |
| ath-miR162a-3p | 1193 | 1335 | 636 | 1707 | 1171 |
| ath-miR162a-5p | 13 | 20 | 2 | 30 | 25 |
| ath-miR164a | 254 | 394 | 292 | 258 | 283 |
| ath-miR164c-5p | 254 | 394 | 292 | 258 | 283 |
| ath-miR165a-3p | 267 | 433 | 350 | 228 | 476 |
| ath-miR165a-5p | 0 | 1 | 0 | 0 | 1 |
| ath-miR166a-3p | 49504 | 77905 | 62121 | 47681 | 76408 |
| ath-miR166a-5p | 0 | 0 | 0 | 1 | 1 |
| ath-miR166e-5p | 0 | 2 | 0 | 0 | 3 |
| ath-miR167a-5p | 1873 | 2530 | 1296 | 958 | 1875 |
| ath-miR167c-5p | 6 | 3 | 3 | 0 | 0 |
| ath-miR167d | 7543 | 10367 | 10205 | 7346 | 11461 |
| ath-miR168a-3p | 1619 | 2344 | 1119 | 1230 | 1532 |
| ath-miR168a-5p | 2248 | 3170 | 2584 | 2513 | 3161 |
| ath-miR169d | 0 | 1 | 0 | 0 | 0 |
| ath-miR170-3p | 1 | 0 | 0 | 0 | 0 |
| ath-miR171a-3p | 1 | 1 | 3 | 1 | 0 |
| ath-miR171b-3p | 340 | 383 | 248 | 141 | 139 |
| ath-miR171c-5p | 97 | 151 | 157 | 97 | 98 |
| ath-miR172a | 4908 | 5507 | 4446 | 2855 | 5070 |
| ath-miR172c | 700 | 717 | 611 | 310 | 588 |
| ath-miR172e-3p | 33 | 25 | 22 | 12 | 25 |
| ath-miR319a | 17 | 21 | 14 | 8 | 14 |
| ath-miR319c | 37 | 51 | 23 | 20 | 27 |
| ath-miR393a-3p | 1 | 0 | 0 | 0 | 0 |
| ath-miR393a-5p | 63 | 114 | 44 | 23 | 59 |
| ath-miR394a | 1171 | 1770 | 1387 | 1059 | 1225 |
| ath-miR395a | 5 | 9 | 48 | 10 | 37 |
| ath-miR395b | 0 | 0 | 4 | 2 | 1 |
| ath-miR396a-3p | 6085 | 8887 | 7970 | 6916 | 8806 |
| ath-miR396a-5p | 20027 | 38132 | 19809 | 17207 | 23121 |
| ath-miR396b-3p | 116 | 265 | 105 | 175 | 83 |
| ath-miR396b-5p | 49860 | 86682 | 39029 | 54170 | 35993 |
| ath-miR397a | 0 | 0 | 0 | 0 | 1 |
| ath-miR398a-3p | 423 | 1512 | 92 | 26 | 109 |
| ath-miR398b-3p | 19 | 58 | 5 | 2 | 7 |
| ath-miR399a | 26 | 31 | 67 | 42 | 48 |
| ath-miR399b | 16 | 61 | 39 | 39 | 28 |
| ath-miR399d | 1 | 0 | 1 | 1 | 2 |
| ath-miR399e | 0 | 1 | 0 | 0 | 0 |
| ath-miR399f | 2 | 0 | 0 | 0 | 1 |
| ath-miR408-3p | 300 | 538 | 203 | 119 | 473 |
| ath-miR5658 | 0 | 0 | 0 | 0 | 1 |
| ath-miR8175 | 11 | 9 | 12 | 28 | 15 |
| atr-miR397a | 0 | 0 | 0 | 0 | 2 |
| atr-miR398 | 6 | 15 | 1 | 1 | 19 |
| atr-miR8577 | 0 | 0 | 0 | 0 | 1 |
| bdi-miR159a-3p | 167 | 229 | 107 | 58 | 138 |
| bdi-miR159a-5p | 1 | 0 | 0 | 0 | 0 |
| bdi-miR162 | 172 | 245 | 68 | 161 | 118 |
| bdi-miR395p-3p | 0 | 0 | 1 | 0 | 0 |
| bna-miR167d | 8379 | 10993 | 10161 | 7307 | 11667 |
| bna-miR168b | 10 | 8 | 6 | 3 | 6 |
| bna-miR169m | 63 | 105 | 78 | 56 | 58 |
| bra-miR408-5p | 300 | 538 | 203 | 119 | 473 |
| cca-miR156b | 4457 | 4817 | 1877 | 987 | 2262 |
| cca-miR396a-3p | 184 | 341 | 158 | 193 | 154 |
| cca-miR396c | 19 | 42 | 23 | 13 | 27 |
| cca-miR6113 | 2 | 18 | 4 | 1 | 10 |
| cln-miR162 | 4 | 1 | 1 | 3 | 1 |
| cme-miR166i | 1762 | 2394 | 1068 | 589 | 1373 |
| cme-miR169k | 0 | 1 | 0 | 0 | 0 |
| cme-miR169q | 3 | 8 | 6 | 2 | 2 |
| cme-miR399d | 1 | 5 | 1 | 3 | 0 |
| cpa-miR166e | 48488 | 76149 | 61269 | 47285 | 75254 |
| csi-miR160 | 155 | 220 | 149 | 143 | 197 |
| ctr-miR171 | 9 | 19 | 10 | 5 | 17 |
| ghr-miR156c | 13 | 17 | 12 | 7 | 15 |
| gma-miR1509a | 8 | 7 | 5 | 7 | 1 |
| gma-miR1509b | 27 | 18 | 26 | 24 | 30 |
| gma-miR1511 | 7668 | 12127 | 10073 | 6256 | 14959 |
| gma-miR1514a-3p | 0 | 0 | 1 | 1 | 0 |
| gma-miR1514a-5p | 0 | 0 | 0 | 1 | 0 |
| gma-miR1525 | 1 | 1 | 1 | 1 | 0 |
| gma-miR1527 | 4 | 9 | 6 | 4 | 1 |
| gma-miR156g | 2 | 4 | 0 | 1 | 0 |
| gma-miR156r | 0 | 1 | 0 | 1 | 0 |
| gma-miR159d | 49 | 112 | 67 | 36 | 233 |
| gma-miR160b | 155 | 220 | 149 | 143 | 197 |
| gma-miR164b | 254 | 394 | 292 | 258 | 283 |
| gma-miR166m | 25 | 64 | 33 | 31 | 38 |
| gma-miR166u | 56789 | 87366 | 67818 | 51704 | 84033 |
| gma-miR167h | 636 | 1009 | 488 | 464 | 745 |
| gma-miR167i | 265 | 496 | 263 | 260 | 432 |
| gma-miR167k | 1 | 0 | 0 | 0 | 0 |
| gma-miR168b | 2093 | 2981 | 2370 | 2399 | 2929 |
| gma-miR169e | 3 | 10 | 13 | 14 | 12 |
| gma-miR171a | 338 | 379 | 243 | 141 | 137 |
| gma-miR171h | 0 | 0 | 0 | 1 | 1 |
| gma-miR171m | 9 | 19 | 10 | 5 | 17 |
| gma-miR171n | 54 | 68 | 36 | 17 | 65 |
| gma-miR171o-5p | 1 | 0 | 0 | 0 | 0 |
| gma-miR172b-5p | 31 | 39 | 45 | 39 | 20 |
| gma-miR172d | 3 | 9 | 6 | 5 | 7 |
| gma-miR172h-5p | 47 | 72 | 66 | 74 | 70 |
| gma-miR2109-5p | 1 | 0 | 0 | 0 | 0 |
| gma-miR2118a-3p | 3 | 4 | 8 | 8 | 10 |
| gma-miR319q | 17 | 21 | 14 | 8 | 14 |
| gma-miR395d | 0 | 0 | 4 | 1 | 1 |
| gma-miR396a-3p | 6036 | 8838 | 7933 | 6891 | 8740 |
| gma-miR396b-3p | 4066 | 7280 | 5534 | 8746 | 3858 |
| gma-miR396d | 7 | 15 | 7 | 17 | 8 |
| gma-miR396h | 20039 | 38162 | 19812 | 17206 | 23123 |
| gma-miR396j | 0 | 1 | 0 | 0 | 0 |
| gma-miR408d | 300 | 538 | 203 | 119 | 473 |
| gma-miR4403 | 1 | 1 | 6 | 1 | 1 |
| gma-miR4415b-5p | 0 | 1 | 0 | 0 | 0 |
| gma-miR4995 | 9 | 13 | 16 | 25 | 14 |
| gma-miR530a | 1 | 0 | 0 | 0 | 1 |
| gma-miR5368 | 97 | 75 | 96 | 111 | 57 |
| gma-miR5770a | 1 | 0 | 0 | 1 | 3 |
| gma-miR5770b | 1 | 0 | 0 | 1 | 3 |
| gra-miR166c | 53 | 95 | 39 | 26 | 37 |
| gra-miR166d | 14 | 43 | 26 | 17 | 32 |
| gra-miR167c | 4752 | 6068 | 3107 | 2550 | 4713 |
| hbr-miR156 | 15 | 21 | 13 | 9 | 12 |
| hbr-miR396a | 0 | 12 | 4 | 4 | 4 |
| hbr-miR6173 | 43 | 49 | 28 | 45 | 34 |
| hbr-miR6483 | 116 | 185 | 152 | 164 | 121 |
| hci-miR156a | 182 | 241 | 69 | 41 | 106 |
| hci-miR164a | 64 | 76 | 39 | 28 | 40 |
| htu-miR171a | 371 | 457 | 276 | 177 | 174 |
| hvu-miR397a | 0 | 0 | 0 | 0 | 2 |
| hvu-miR5049c | 0 | 0 | 1 | 2 | 0 |
| lja-miR171c | 0 | 2 | 4 | 1 | 1 |
| lja-miR7534 | 1 | 0 | 0 | 0 | 0 |
| lus-miR159b | 33767 | 44222 | 18859 | 13760 | 22823 |
| lus-miR172j | 1 | 4 | 3 | 4 | 5 |
| lus-miR398f | 0 | 5 | 1 | 0 | 2 |
| mdm-miR156ad | 8 | 7 | 10 | 11 | 11 |
| mdm-miR159a | 28 | 31 | 21 | 6 | 11 |
| mdm-miR164a | 2 | 3 | 0 | 0 | 0 |
| mdm-miR391 | 2 | 0 | 1 | 1 | 0 |
| mdm-miR396a | 2412 | 4971 | 2518 | 2058 | 2882 |
| mes-miR166i | 70 | 126 | 100 | 84 | 113 |
| mes-miR171d | 0 | 3 | 1 | 0 | 1 |
| mes-miR172c | 30 | 33 | 44 | 38 | 18 |
| mes-miR397 | 0 | 0 | 0 | 0 | 2 |
| mtr-miR1507-5p | 1 | 0 | 1 | 0 | 0 |
| mtr-miR1510a-3p | 4 | 10 | 9 | 2 | 8 |
| mtr-miR1510a-5p | 6393 | 9552 | 7050 | 6076 | 7030 |
| mtr-miR156b-3p | 28 | 40 | 30 | 24 | 21 |
| mtr-miR156c-3p | 516 | 827 | 604 | 511 | 562 |
| mtr-miR156d-3p | 2 | 6 | 1 | 5 | 2 |
| mtr-miR156g-3p | 7 | 15 | 10 | 4 | 10 |
| mtr-miR156g-5p | 1577 | 2814 | 1098 | 1007 | 868 |
| mtr-miR156i-3p | 15 | 13 | 26 | 21 | 27 |
| mtr-miR156j | 84 | 104 | 76 | 57 | 72 |
| mtr-miR160f | 4 | 7 | 4 | 7 | 4 |
| mtr-miR166b | 4641 | 7186 | 5716 | 3836 | 5967 |
| mtr-miR167b-3p | 80 | 46 | 56 | 85 | 199 |
| mtr-miR169e-5p | 1 | 0 | 0 | 1 | 1 |
| mtr-miR169h | 340 | 490 | 528 | 597 | 366 |
| mtr-miR169j | 38 | 69 | 78 | 49 | 51 |
| mtr-miR169k | 0 | 1 | 0 | 2 | 3 |
| mtr-miR171a | 19 | 26 | 35 | 28 | 33 |
| mtr-miR171b | 316 | 545 | 351 | 227 | 549 |
| mtr-miR171c | 18 | 21 | 6 | 6 | 4 |
| mtr-miR172a | 100 | 165 | 121 | 111 | 64 |
| mtr-miR172d-5p | 11 | 28 | 11 | 17 | 23 |
| mtr-miR2088-5p | 0 | 0 | 2 | 3 | 1 |
| mtr-miR2118 | 1167 | 1747 | 3972 | 2777 | 4826 |
| mtr-miR2119 | 39 | 143 | 20 | 12 | 26 |
| mtr-miR2199 | 21 | 29 | 39 | 28 | 21 |
| mtr-miR2586a | 6 | 19 | 4 | 10 | 9 |
| mtr-miR2593e | 0 | 1 | 0 | 0 | 1 |
| mtr-miR2604 | 0 | 0 | 0 | 0 | 1 |
| mtr-miR2606a | 0 | 0 | 0 | 0 | 1 |
| mtr-miR2606c | 1 | 3 | 3 | 5 | 3 |
| mtr-miR2614 | 0 | 2 | 0 | 1 | 2 |
| mtr-miR2630a | 0 | 1 | 1 | 0 | 0 |
| mtr-miR2645 | 1 | 0 | 0 | 1 | 8 |
| mtr-miR2666 | 0 | 0 | 0 | 0 | 2 |
| mtr-miR2673a | 0 | 1 | 0 | 0 | 1 |
| mtr-miR2678 | 12 | 14 | 6 | 7 | 13 |
| mtr-miR319a-3p | 17 | 21 | 14 | 8 | 14 |
| mtr-miR319a-5p | 0 | 1 | 2 | 4 | 2 |
| mtr-miR393b-3p | 101 | 117 | 81 | 63 | 38 |
| mtr-miR395a | 15 | 50 | 279 | 66 | 274 |
| mtr-miR395g | 0 | 0 | 3 | 2 | 2 |
| mtr-miR395h | 1 | 0 | 8 | 2 | 4 |
| mtr-miR398a-5p | 406 | 1996 | 207 | 63 | 196 |
| mtr-miR399k | 0 | 1 | 3 | 1 | 0 |
| mtr-miR4414a-5p | 1 | 7 | 1 | 0 | 1 |
| mtr-miR482-3p | 412 | 455 | 565 | 322 | 445 |
| mtr-miR5037a | 22 | 81 | 95 | 87 | 64 |
| mtr-miR5205a | 0 | 0 | 0 | 1 | 0 |
| mtr-miR5205b | 10 | 13 | 7 | 11 | 13 |
| mtr-miR5205c | 5 | 10 | 2 | 3 | 8 |
| mtr-miR5206a | 0 | 2 | 0 | 0 | 0 |
| mtr-miR5208a | 37 | 66 | 39 | 56 | 71 |
| mtr-miR5208d | 1 | 0 | 0 | 0 | 0 |
| mtr-miR5211 | 91 | 55 | 31 | 29 | 1337 |
| mtr-miR5213-5p | 91940 | 122914 | 189108 | 112265 | 177534 |
| mtr-miR5217 | 0 | 0 | 1 | 0 | 0 |
| mtr-miR5231 | 2 | 0 | 0 | 0 | 1 |
| mtr-miR5232 | 400 | 556 | 236 | 123 | 320 |
| mtr-miR5234 | 3 | 3 | 0 | 2 | 7 |
| mtr-miR5235a | 1 | 2 | 0 | 1 | 0 |
| mtr-miR5237 | 11 | 5 | 4 | 2 | 5 |
| mtr-miR5239 | 106733 | 141960 | 160387 | 138313 | 151062 |
| mtr-miR5241a | 2 | 1 | 1 | 0 | 7 |
| mtr-miR5245 | 75 | 83 | 44 | 41 | 51 |
| mtr-miR5248 | 9 | 10 | 9 | 5 | 16 |
| mtr-miR5255 | 3 | 6 | 2 | 6 | 9 |
| mtr-miR5256 | 73 | 69 | 105 | 69 | 280 |
| mtr-miR5261 | 31 | 28 | 0 | 0 | 0 |
| mtr-miR5267a | 22 | 35 | 28 | 22 | 36 |
| mtr-miR5269a | 0 | 0 | 1 | 0 | 0 |
| mtr-miR5269b | 4 | 4 | 3 | 2 | 6 |
| mtr-miR5272a | 126 | 131 | 116 | 121 | 115 |
| mtr-miR5272f | 0 | 1 | 2 | 0 | 1 |
| mtr-miR5277 | 1 | 0 | 0 | 0 | 2 |
| mtr-miR5281b | 1 | 0 | 0 | 1 | 0 |
| mtr-miR5286a | 66 | 85 | 61 | 80 | 98 |
| mtr-miR5286b | 28 | 52 | 44 | 54 | 66 |
| mtr-miR5287a | 16 | 17 | 24 | 30 | 14 |
| mtr-miR5287b | 33 | 31 | 64 | 40 | 47 |
| mtr-miR5290 | 2 | 1 | 1 | 0 | 3 |
| mtr-miR5291a | 4 | 5 | 1 | 0 | 1 |
| mtr-miR5299 | 69 | 95 | 88 | 96 | 118 |
| mtr-miR5559-3p | 0 | 1 | 0 | 0 | 0 |
| mtr-miR5559-5p | 1381 | 5859 | 1616 | 663 | 1058 |
| mtr-miR5561-3p | 5 | 9 | 17 | 4 | 10 |
| mtr-miR5741a | 0 | 0 | 0 | 1 | 0 |
| mtr-miR5743a | 1 | 1 | 2 | 0 | 4 |
| mtr-miR5745a | 2 | 1 | 2 | 3 | 1 |
| mtr-miR5753 | 3 | 6 | 2 | 1 | 2 |
| mtr-miR7696a-3p | 18003 | 24931 | 32461 | 24652 | 31133 |
| mtr-miR7696a-5p | 17831 | 24711 | 32215 | 24432 | 30865 |
| mtr-miR7696c-3p | 18008 | 24931 | 32471 | 24660 | 31144 |
| mtr-miR7696c-5p | 1919 | 2789 | 3001 | 1992 | 2845 |
| mtr-miR7696d-5p | 1915 | 2785 | 2996 | 1993 | 2841 |
| mtr-miR7701-3p | 3 | 11 | 6 | 6 | 9 |
| mtr-miR7701-5p | 0 | 0 | 2 | 1 | 0 |
| nta-miR156f | 9 | 14 | 16 | 12 | 10 |
| nta-miR172b | 4 | 2 | 3 | 1 | 2 |
| nta-miR397 | 0 | 0 | 0 | 0 | 1 |
| osa-miR1436 | 0 | 0 | 3 | 0 | 0 |
| osa-miR156b-3p | 3 | 1 | 1 | 0 | 0 |
| osa-miR156f-3p | 0 | 1 | 0 | 0 | 0 |
| osa-miR156k | 12 | 11 | 14 | 6 | 9 |
| osa-miR156l-5p | 3 | 4 | 3 | 2 | 2 |
| osa-miR159a.1 | 49182 | 58729 | 24735 | 18016 | 30373 |
| osa-miR159c | 35 | 36 | 14 | 11 | 24 |
| osa-miR159d | 24 | 27 | 8 | 9 | 11 |
| osa-miR159e | 22 | 26 | 8 | 9 | 11 |
| osa-miR159f | 1071 | 1559 | 1393 | 1140 | 1362 |
| osa-miR160e-5p | 53 | 75 | 43 | 27 | 38 |
| osa-miR162b | 4 | 5 | 3 | 6 | 1 |
| osa-miR164d | 339 | 461 | 321 | 283 | 317 |
| osa-miR164e | 64 | 76 | 39 | 28 | 40 |
| osa-miR166b-5p | 31 | 44 | 35 | 46 | 28 |
| osa-miR166d-5p | 1308 | 2063 | 1586 | 1533 | 1846 |
| osa-miR166e-3p | 40 | 77 | 52 | 37 | 62 |
| osa-miR166g-3p | 6168 | 9417 | 7537 | 5496 | 7387 |
| osa-miR166h-5p | 201 | 213 | 179 | 116 | 166 |
| osa-miR166i-3p | 5 | 6 | 9 | 4 | 6 |
| osa-miR166k-3p | 1 | 1 | 1 | 0 | 0 |
| osa-miR166m | 27513 | 32413 | 14327 | 9727 | 21747 |
| osa-miR167d-5p | 8584 | 11852 | 12515 | 8532 | 13940 |
| osa-miR171b | 371 | 457 | 276 | 177 | 175 |
| osa-miR171i-3p | 9 | 19 | 10 | 5 | 17 |
| osa-miR172c | 3 | 2 | 1 | 2 | 4 |
| osa-miR1863a | 1 | 0 | 0 | 0 | 0 |
| osa-miR1873 | 1 | 0 | 0 | 0 | 0 |
| osa-miR393a | 63 | 114 | 44 | 23 | 59 |
| osa-miR395b | 14 | 42 | 224 | 56 | 245 |
| osa-miR395c | 0 | 0 | 1 | 0 | 0 |
| osa-miR396a-3p | 878 | 1196 | 558 | 499 | 799 |
| osa-miR396c-3p | 0 | 0 | 1 | 0 | 0 |
| osa-miR396e-5p | 1 | 0 | 0 | 0 | 0 |
| osa-miR396f-5p | 1 | 0 | 0 | 0 | 0 |
| osa-miR397b | 0 | 0 | 0 | 0 | 2 |
| osa-miR398b | 2200 | 3281 | 840 | 737 | 2787 |
| osa-miR399e | 1 | 7 | 11 | 11 | 10 |
| osa-miR399j | 1 | 4 | 1 | 3 | 0 |
| osa-miR408-3p | 24 | 41 | 15 | 13 | 39 |
| osa-miR5523 | 0 | 1 | 0 | 1 | 0 |
| osa-miR5532 | 0 | 0 | 1 | 0 | 0 |
| osa-miR5538 | 1 | 0 | 1 | 1 | 0 |
| pde-miR159 | 49 | 79 | 76 | 71 | 69 |
| ppe-miR1511-3p | 1 | 2 | 3 | 1 | 0 |
| ppe-miR169e-5p | 0 | 1 | 0 | 0 | 0 |
| ppe-miR393a | 63 | 114 | 44 | 23 | 59 |
| ppe-miR395a-5p | 0 | 0 | 1 | 0 | 0 |
| ppe-miR396a | 4117 | 7400 | 3319 | 2917 | 3738 |
| ppe-miR398b | 11 | 21 | 5 | 1 | 12 |
| ppt-miR160b | 1 | 0 | 0 | 0 | 0 |
| ppt-miR160c | 0 | 0 | 1 | 0 | 0 |
| ppt-miR160d | 0 | 0 | 1 | 0 | 0 |
| ppt-miR166j | 190 | 315 | 208 | 192 | 242 |
| ppt-miR166m | 0 | 1 | 1 | 0 | 1 |
| ppt-miR167 | 2 | 2 | 0 | 4 | 0 |
| ppt-miR171a | 14 | 12 | 10 | 5 | 4 |
| ppt-miR171b | 14 | 12 | 10 | 5 | 4 |
| ppt-miR319a | 403 | 603 | 211 | 95 | 180 |
| ppt-miR408b | 300 | 540 | 204 | 121 | 474 |
| ppt-miR894 | 0 | 0 | 1 | 1 | 0 |
| pta-miR159a | 38 | 37 | 24 | 8 | 18 |
| pta-miR159c | 28 | 31 | 21 | 6 | 11 |
| pta-miR319 | 406 | 608 | 218 | 100 | 181 |
| ptc-miR156k | 0 | 4 | 0 | 2 | 5 |
| ptc-miR160g | 0 | 1 | 0 | 0 | 0 |
| ptc-miR160h | 9 | 8 | 8 | 5 | 11 |
| ptc-miR164f | 2 | 3 | 0 | 0 | 0 |
| ptc-miR166n | 4255 | 6504 | 4364 | 3048 | 4698 |
| ptc-miR166p | 7 | 25 | 16 | 16 | 25 |
| ptc-miR167f-5p | 4753 | 6069 | 3107 | 2549 | 4712 |
| ptc-miR167h-5p | 11 | 10 | 10 | 5 | 13 |
| ptc-miR171c | 117 | 154 | 87 | 86 | 64 |
| ptc-miR171k | 0 | 0 | 1 | 0 | 0 |
| ptc-miR171l-3p | 2 | 6 | 6 | 2 | 6 |
| ptc-miR172b-5p | 3 | 7 | 5 | 6 | 9 |
| ptc-miR172i | 35 | 65 | 34 | 31 | 21 |
| ptc-miR319e | 17 | 21 | 14 | 8 | 14 |
| ptc-miR393a-3p | 206 | 285 | 163 | 140 | 176 |
| ptc-miR396e-3p | 4063 | 7276 | 5531 | 8742 | 3856 |
| ptc-miR396f | 321 | 501 | 586 | 298 | 334 |
| ptc-miR396g-3p | 0 | 1 | 0 | 0 | 1 |
| ptc-miR396g-5p | 201 | 331 | 162 | 170 | 137 |
| ptc-miR397b | 0 | 0 | 0 | 1 | 2 |
| ptc-miR399d | 1 | 1 | 1 | 1 | 3 |
| ptc-miR6478 | 348 | 389 | 262 | 354 | 303 |
| pvu-miR2118 | 3 | 4 | 8 | 8 | 10 |
| rgl-miR5139 | 0 | 0 | 0 | 1 | 0 |
| rgl-miR5141 | 6 | 3 | 4 | 3 | 1 |
| sbi-miR164c | 0 | 1 | 0 | 0 | 0 |
| sbi-miR172b | 28 | 24 | 18 | 10 | 22 |
| sbi-miR172f | 29 | 57 | 32 | 25 | 17 |
| sbi-miR6224a-5p | 0 | 0 | 0 | 0 | 2 |
| sly-miR156e-5p | 9 | 17 | 12 | 7 | 10 |
| sly-miR166c-5p | 0 | 0 | 0 | 0 | 1 |
| sly-miR167b-5p | 7 | 20 | 12 | 12 | 16 |
| sly-miR168a-3p | 5 | 2 | 3 | 1 | 4 |
| sly-miR171d | 117 | 154 | 87 | 86 | 64 |
| sly-miR4376 | 9 | 13 | 11 | 8 | 10 |
| sly-miR482a | 274 | 450 | 572 | 391 | 477 |
| smo-miR171b | 883 | 1349 | 1014 | 579 | 549 |
| smo-miR396 | 6 | 27 | 12 | 4 | 9 |
| sof-miR159c | 28 | 31 | 21 | 6 | 11 |
| sof-miR159e | 15 | 26 | 8 | 9 | 8 |
| ssp-miR1128 | 2 | 3 | 0 | 1 | 3 |
| stu-miR156f-3p | 0 | 1 | 0 | 0 | 0 |
| stu-miR156f-5p | 4456 | 4817 | 1877 | 987 | 2262 |
| stu-miR156g-3p | 513 | 818 | 597 | 504 | 552 |
| stu-miR171b-3p | 9 | 19 | 10 | 5 | 17 |
| stu-miR171d-5p | 13 | 27 | 14 | 10 | 8 |
| stu-miR172a-5p | 9 | 13 | 8 | 11 | 12 |
| stu-miR172c-3p | 48 | 80 | 54 | 38 | 56 |
| stu-miR398a-3p | 6 | 2 | 0 | 4 | 6 |
| stu-miR4376-5p | 8 | 13 | 10 | 8 | 10 |
| tae-miR1120a | 0 | 0 | 0 | 0 | 1 |
| tae-miR1122c-3p | 0 | 0 | 1 | 0 | 0 |
| tae-miR1128 | 0 | 1 | 0 | 0 | 1 |
| tae-miR167b | 0 | 2 | 0 | 0 | 0 |
| tae-miR395b | 15 | 42 | 225 | 56 | 249 |
| tcc-miR172d | 34 | 77 | 41 | 36 | 32 |
| tcc-miR399f | 0 | 0 | 0 | 0 | 1 |
| vvi-miR156e | 56 | 81 | 69 | 41 | 61 |
| vvi-miR156h | 2 | 5 | 3 | 0 | 4 |
| vvi-miR166a | 3820 | 6033 | 4218 | 2925 | 4491 |
| vvi-miR167c | 1484 | 1988 | 840 | 677 | 1331 |
| vvi-miR169l | 0 | 1 | 0 | 0 | 0 |
| vvi-miR169o | 0 | 1 | 0 | 0 | 0 |
| vvi-miR171f | 117 | 154 | 87 | 86 | 64 |
| vvi-miR171h | 0 | 0 | 1 | 0 | 0 |
| vvi-miR172a | 30 | 38 | 45 | 39 | 19 |
| vvi-miR172b | 30 | 38 | 45 | 39 | 19 |
| vvi-miR3630-3p | 0 | 1 | 1 | 0 | 1 |
| vvi-miR396a | 9496 | 17485 | 6496 | 5586 | 7913 |
| vvi-miR396b | 49165 | 85135 | 38317 | 53249 | 35276 |
| vvi-miR399g | 1 | 0 | 0 | 0 | 1 |
| vvi-miR399i | 0 | 0 | 1 | 0 | 1 |
| zma-miR156k-5p | 32 | 59 | 38 | 28 | 39 |
| zma-miR159c-5p | 1 | 0 | 0 | 0 | 0 |
| zma-miR159h-3p | 1 | 3 | 0 | 0 | 5 |
| zma-miR162-3p | 1170 | 1306 | 621 | 1678 | 1150 |
| zma-miR164h-5p | 41 | 51 | 31 | 21 | 26 |
| zma-miR166h-3p | 48674 | 76886 | 61863 | 47476 | 75915 |
| zma-miR171a-3p | 1 | 1 | 3 | 1 | 0 |
| zma-miR171b-3p | 337 | 378 | 243 | 141 | 137 |
| zma-miR171c-3p | 0 | 1 | 1 | 2 | 3 |
| zma-miR396g-3p | 20011 | 38099 | 19797 | 17192 | 23087 |
| zma-miR396g-5p | 314 | 526 | 299 | 305 | 371 |
| zma-miR398a-3p | 53 | 67 | 8 | 7 | 46 |

**Table S4 The reads of the novel predicted miRNAs at the five genotypes.**

| **miRNA** | **N46** | **N47** | **N48** | **N49** | **RPh** |
| --- | --- | --- | --- | --- | --- |
| novel_10 | 5960 | 9669 | 6790 | 4268 | 7957 |
| novel_100 | 32 | 49 | 53 | 33 | 46 |
| novel_101 | 10 | 14 | 16 | 14 | 48 |
| novel_102 | 15 | 22 | 27 | 16 | 34 |
| novel_104 | 2 | 7 | 23 | 5 | 26 |
| novel_107 | 39 | 35 | 26 | 9 | 17 |
| novel_108 | 11 | 9 | 21 | 18 | 22 |
| novel_111 | 29 | 29 | 27 | 11 | 16 |
| novel_112 | 13 | 23 | 9 | 11 | 15 |
| novel_113 | 0 | 18 | 26 | 1 | 6 |
| novel_114 | 6 | 3 | 20 | 9 | 37 |
| novel_115 | 3 | 4 | 2 | 2 | 4 |
| novel_116 | 6 | 15 | 18 | 6 | 22 |
| novel_118 | 11 | 8 | 8 | 30 | 13 |
| novel_119 | 4 | 11 | 13 | 14 | 10 |
| novel_120 | 3 | 9 | 9 | 8 | 10 |
| novel_121 | 0 | 11 | 6 | 3 | 4 |
| novel_122 | 11 | 11 | 16 | 3 | 12 |
| novel_123 | 9 | 15 | 13 | 3 | 4 |
| novel_124 | 2 | 7 | 4 | 4 | 6 |
| novel_126 | 2 | 5 | 3 | 3 | 4 |
| novel_127 | 4 | 2 | 9 | 11 | 5 |
| novel_128 | 3 | 4 | 2 | 0 | 3 |
| novel_129 | 15 | 14 | 14 | 13 | 20 |
| novel_13 | 4838 | 5353 | 5703 | 3928 | 5247 |
| novel_130 | 10 | 12 | 18 | 11 | 11 |
| novel_132 | 0 | 2 | 4 | 0 | 1 |
| novel_133 | 15 | 29 | 17 | 20 | 37 |
| novel_135 | 103 | 139 | 95 | 100 | 70 |
| novel_136 | 1 | 2 | 3 | 4 | 3 |
| novel_137 | 63 | 90 | 96 | 63 | 124 |
| novel_139 | 8 | 16 | 14 | 18 | 14 |
| novel_142 | 7 | 12 | 16 | 14 | 12 |
| novel_143 | 2 | 1 | 1 | 0 | 0 |
| novel_144 | 272 | 312 | 307 | 287 | 436 |
| novel_145 | 6 | 11 | 11 | 6 | 14 |
| novel_146 | 0 | 0 | 0 | 0 | 1 |
| novel_147 | 23 | 18 | 30 | 31 | 26 |
| novel_17 | 1 | 1 | 4591 | 3939 | 4130 |
| novel_19 | 1615 | 2308 | 2688 | 2274 | 2120 |
| novel_20 | 1992 | 2445 | 1680 | 1193 | 1635 |
| novel_21 | 1274 | 1725 | 2108 | 1058 | 2467 |
| novel_24 | 1203 | 1827 | 1676 | 1206 | 1888 |
| novel_26 | 729 | 1129 | 1847 | 922 | 2788 |
| novel_3 | 4 | 13 | 5 | 5 | 3 |
| novel_30 | 512 | 665 | 820 | 542 | 812 |
| novel_32 | 1153 | 2 | 1031 | 7 | 1036 |
| novel_34 | 370 | 557 | 374 | 174 | 301 |
| novel_43 | 14 | 13 | 16 | 3 | 12 |
| novel_50 | 181 | 202 | 182 | 229 | 161 |
| novel_55 | 380 | 551 | 339 | 283 | 388 |
| novel_56 | 288 | 418 | 448 | 313 | 358 |
| novel_6 | 9061 | 18291 | 10916 | 6560 | 7636 |
| novel_61 | 212 | 279 | 310 | 194 | 275 |
| novel_62 | 173 | 253 | 250 | 157 | 219 |
| novel_64 | 228 | 388 | 559 | 245 | 257 |
| novel_67 | 110 | 109 | 58 | 64 | 298 |
| novel_68 | 145 | 200 | 263 | 214 | 426 |
| novel_75 | 94 | 199 | 312 | 207 | 205 |
| novel_76 | 183 | 179 | 76 | 99 | 222 |
| novel_77 | 82 | 6 | 26 | 463 | 359 |
| novel_78 | 73 | 123 | 216 | 144 | 188 |
| novel_79 | 44 | 134 | 77 | 70 | 64 |
| novel_80 | 19 | 31 | 34 | 25 | 58 |
| novel_81 | 99 | 89 | 60 | 89 | 104 |
| novel_83 | 128 | 139 | 134 | 181 | 105 |
| novel_85 | 41 | 52 | 57 | 53 | 60 |
| novel_87 | 46 | 124 | 139 | 145 | 67 |
| novel_89 | 71 | 112 | 164 | 74 | 137 |
| novel_9 | 5955 | 8607 | 7241 | 4522 | 6248 |
| novel_93 | 52 | 93 | 64 | 51 | 65 |
| novel_95 | 79 | 98 | 66 | 59 | 93 |
| novel_96 | 35 | 77 | 49 | 30 | 26 |
| novel_97 | 56 | 83 | 84 | 52 | 76 |
| novel_98 | 19 | 8 | 87 | 9 | 56 |
| novel_99 | 28 | 40 | 41 | 26 | 22 |

**Table S5 Detailed information of novel miRNAs in *M. albus***

**Table S6 Summary of the transcriptome sequencing in *M. albus***

| **Genotype** | **Raw reads** | **Clean reads** | **Total transcripts** | **Average length** | **N50 length** | **N90 length** |
| --- | --- | --- | --- | --- | --- | --- |
| N46 | 32939751 | 30532020 | 154458 | 1003 | 1782 | 383 |
| N47 | 31176000 | 28785103 |  |  |  |  |
| N48 | 32518646 | 30067146 |  |  |  |  |
| N49 | 31470600 | 29041739 |  |  |  |  |
| RPh | 35446843 | 32634517 |  |  |  |  |

**Table S7 Detailed information on the different miRNAs in four compasion groups (N47 vs N46, N48 vs N46, N49 vs N47 and N49 vs N48).**

| **Compasion group** | **miRNA** | **log2.Fold_change.** |
| --- | --- | --- |
| N47vsN46 | mtr-miR5559-5p | 1.5615 |
|  | novel_32 | -9.6946 |
|  | mtr-miR398a-5p | 1.7742 |
|  | ahy-miR398 | 1.3217 |
|  | ath-miR398a-3p | 1.3143 |
|  | novel_77 | -4.296 |
|  | mtr-miR2119 | 1.3511 |
|  | mtr-miR5211 | -1.2498 |
|  | mtr-miR167b-3p | -1.3218 |
|  | ahy-miR156a | -1.6546 |
|  | novel_79 | 1.0833 |
|  | mtr-miR5037a | 1.357 |
|  | novel_113 | 4.367 |
|  | ath-miR399b | 1.4073 |
| N48vsN46 | ath-miR159b-3p | -1.0799 |
|  | ath-miR159c | -1.3533 |
|  | lus-miR159b | -1.0649 |
|  | novel_17 | 11.94 |
|  | osa-miR159a.1 | -1.2161 |
|  | osa-miR166m | -1.1659 |
|  | cca-miR156b | -1.4721 |
|  | stu-miR156f-5p | -1.4718 |
|  | mtr-miR2118 | 1.5426 |
|  | osa-miR398b | -1.6135 |
|  | novel_26 | 1.1167 |
|  | vvi-miR167c | -1.0455 |
|  | ath-miR398a-3p | -2.4254 |
|  | ahy-miR398 | -2.4309 |
|  | ath-miR162a-3p | -1.132 |
|  | zma-miR162-3p | -1.1383 |
|  | mtr-miR395a | 3.9927 |
|  | ata-miR395b-3p | 3.7819 |
|  | osa-miR395b | 3.7755 |
|  | tae-miR395b | 3.6824 |
|  | mtr-miR398a-5p | -1.1963 |
|  | ppt-miR319a | -1.158 |
|  | novel_64 | 1.0693 |
|  | pta-miR319 | -1.1216 |
|  | novel_75 | 1.5063 |
|  | hci-miR156a | -1.6238 |
|  | bdi-miR162 | -1.5633 |
|  | novel_76 | -1.4923 |
|  | novel_78 | 1.3406 |
|  | zma-miR398a-3p | -2.9524 |
|  | mtr-miR5211 | -1.7781 |
|  | novel_77 | -1.8816 |
|  | mtr-miR5037a | 1.8859 |
|  | novel_98 | 1.9705 |
|  | mtr-miR5261 | -5.6847 |
|  | novel_87 | 1.3709 |
|  | ath-miR395a | 3.0385 |
|  | novel_67 | -1.1479 |
|  | novel_113 | 5.2065 |
|  | ahy-miR156a | -1.8412 |
|  | novel_104 | 3.2991 |
|  | osa-miR159c | -1.5464 |
|  | ath-miR399a | 1.1412 |
|  | osa-miR159d | -1.8095 |
|  | ath-miR398b-3p | -2.1505 |
|  | aly-miR162a-5p | -2.9249 |
|  | ath-miR162a-5p | -2.9249 |
| N49vsN47 | ath-miR159c | -1.4154 |
|  | mtr-miR5559-5p | -2.428 |
|  | novel_17 | 12.659 |
|  | osa-miR166m | -1.0209 |
|  | mtr-miR398a-5p | -4.27 |
|  | cca-miR156b | -1.5714 |
|  | stu-miR156f-5p | -1.5714 |
|  | ath-miR398a-3p | -5.1462 |
|  | ahy-miR398 | -5.1433 |
|  | mtr-miR2118 | 1.3843 |
|  | osa-miR398b | -1.4388 |
|  | novel_77 | 6.9855 |
|  | cme-miR166i | -1.3075 |
|  | ath-miR162a-3p | 1.0702 |
|  | zma-miR162-3p | 1.0772 |
|  | ppt-miR319a | -1.9505 |
|  | pta-miR319 | -1.8884 |
|  | mtr-miR5232 | -1.4608 |
|  | ath-miR408-3p | -1.461 |
|  | bra-miR408-5p | -1.461 |
|  | gma-miR408d | -1.461 |
|  | ppt-miR408b | -1.4423 |
|  | mtr-miR169h | 1.0006 |
|  | hci-miR156a | -1.8397 |
|  | mtr-miR2119 | -2.8593 |
|  | bdi-miR159a-3p | -1.2656 |
|  | ath-miR398b-3p | -4.1424 |
|  | novel_83 | 1.0965 |
|  | ata-miR393-5p | -1.9848 |
|  | zma-miR398a-3p | -2.5431 |
|  | mtr-miR167b-3p | 1.6015 |
|  | ath-miR393a-5p | -1.5937 |
|  | osa-miR393a | -1.5937 |
|  | ppe-miR393a | -1.5937 |
|  | gma-miR5368 | 1.2812 |
|  | novel_118 | 2.6225 |
|  | mtr-miR5261 | -4.9462 |
|  | ath-miR8175 | 2.3531 |
|  | gra-miR166c | -1.1538 |
|  | ppe-miR398b | -3.6767 |
|  | gma-miR171n | -1.2844 |
|  | mtr-miR395a | 1.1162 |
|  | ata-miR395b-3p | 1.1307 |
|  | osa-miR395b | 1.1307 |
|  | tae-miR395b | 1.1307 |
|  | cca-miR6113 | -3.4543 |
|  | novel_113 | -3.4543 |
|  | mtr-miR5287a | 1.5351 |
|  | novel_147 | 1.4999 |
|  | smo-miR396 | -2.0393 |
|  | gma-miR4995 | 1.659 |
|  | novel_127 | 3.1751 |
| N49vsN48 | gma-miR396b-3p | 1.1199 |
|  | ptc-miR396e-3p | 1.12 |
|  | novel_32 | -6.7429 |
|  | ath-miR162a-3p | 1.8839 |
|  | zma-miR162-3p | 1.8936 |
|  | novel_77 | 4.614 |
|  | mtr-miR395a | -1.6202 |
|  | ata-miR395b-3p | -1.5469 |
|  | tae-miR395b | -1.5469 |
|  | osa-miR395b | -1.5405 |
|  | bdi-miR162 | 1.703 |
|  | novel_98 | -2.8135 |
|  | mtr-miR398a-5p | -1.2567 |
|  | ama-miR396-3p | 1.1965 |
|  | ath-miR396b-3p | 1.1965 |
|  | aly-miR162a-5p | 4.3664 |
|  | ath-miR162a-5p | 4.3664 |
|  | ath-miR398a-3p | -1.3636 |
|  | ahy-miR398 | -1.3478 |
|  | novel_113 | -4.2409 |
|  | ath-miR395a | -1.8035 |
|  | novel_118 | 2.3664 |
|  | mtr-miR167b-3p | 1.0616 |
|  | novel_81 | 1.0284 |
|  | ath-miR8175 | 1.6819 |

**Table S8** **a. The selected miRNAs fold change and p-value in N48 vs N46**

| **miRNA name** | **Fold change** | **p.value** | **Comparison** |
| --- | --- | --- | --- |
| ahy-miR156a | 0.279096218 | 4.42E-05 | N48 vs N46 |
| ama-miR396-3p | 0.774732606 | 0.084045 | N48 vs N46 |
| ata-miR395b-3p | 13.75545648 | 8.38E-34 | N48 vs N46 |
| ath-miR159b-3p | 0.473073984 | 0 | N48 vs N46 |
| ath-miR159c | 0.391403813 | 0 | N48 vs N46 |
| ath-miR395a | 8.21659267 | 3.21E-07 | N48 vs N46 |
| ath-miR396b-3p | 0.774732606 | 0.084045 | N48 vs N46 |
| ath-miR8175 | 0.933703713 | 0.86855 | N48 vs N46 |
| bdi-miR159a-3p | 0.548387859 | 1.21E-05 | N48 vs N46 |
| cca-miR6113 | 1.71179014 | 0.58274 | N48 vs N46 |
| gma-miR396a-3p | 1.124886612 | 2.82E-08 | N48 vs N46 |
| hci-miR156a | 0.324487691 | 9.45E-14 | N48 vs N46 |
| lus-miR159b | 0.478020704 | 0 | N48 vs N46 |
| mtr-miR395a | 15.9196483 | 5.92E-43 | N48 vs N46 |
| mtr-miR5286b | 1.344977967 | 0.2936 | N48 vs N46 |
| mtr-miR5290 | 0.427947283 | 0.52686 | N48 vs N46 |
| mtr-miR5559-5p | 1.001539778 | 0.83521 | N48 vs N46 |
| mtr-miR5561-3p | 2.910043237 | 0.046589 | N48 vs N46 |
| osa-miR159d | 0.285298357 | 0.0035474 | N48 vs N46 |
| osa-miR398b | 0.326796299 | 1.08E-146 | N48 vs N46 |
| ppe-miR398b | 0.389043214 | 0.10741 | N48 vs N46 |
| ppt-miR319a | 0.448123721 | 7.94E-18 | N48 vs N46 |
| pta-miR159c | 0.641921302 | 0.16652 | N48 vs N46 |
| pta-miR319 | 0.459569274 | 3.84E-17 | N48 vs N46 |
| ptc-miR171c | 0.636434796 | 0.0040341 | N48 vs N46 |
| sof-miR159c | 0.641921302 | 0.16652 | N48 vs N46 |
| stu-miR156f-5p | 0.360528511 | 8.11E-259 | N48 vs N46 |
| tae-miR395b | 12.83842605 | 3.38E-33 | N48 vs N46 |

**Table S8** **b. The selected target genes fold change and p-value in N48 vs N46**

| **Gene name** | **Fold change** | **p.value** | **Comparison** |
| --- | --- | --- | --- |
| c10498_g1 | 0 | 1.22E-11 | N48 vs N46 |
| c14529_g1 | 0 | 0.055603 | N48 vs N46 |
| c18626_g1 | 0.003454694 | 1.67E-26 | N48 vs N46 |
| c20788_g1 | 0 | 0.00000792 | N48 vs N46 |
| c24424_g1 | 65.71365535 | 0.0000162 | N48 vs N46 |
| c27695_g1 | 0.307200451 | 0.054372 | N48 vs N46 |
| c29588_g1 | 0.010259581 | 8.7E-18 | N48 vs N46 |
| c29993_g1 | 0.05022784 | 1.87E-19 | N48 vs N46 |
| c30493_g1 | 0.001333405 | 0.0000169 | N48 vs N46 |
| c30598_g2 | 37.90407333 | 0.000000217 | N48 vs N46 |
| c32079_g1 | 0.024909887 | 1.66E-22 | N48 vs N46 |
| c33771_g1 | 0.655342675 | 0.39354 | N48 vs N46 |
| c33813_g2 | 1.518792173 | 0.00057959 | N48 vs N46 |
| c33816_g1 | 1.155674677 | 0.69859 | N48 vs N46 |
| c34206_g4 | 1.061423434 | 0.66868 | N48 vs N46 |
| c34784_g1 | 0.187687533 | 7.54E-18 | N48 vs N46 |
| c35097_g1 | 0 | 1.35E-37 | N48 vs N46 |
| c35242_g1 | 227.1046456 | 6.62E-33 | N48 vs N46 |
| c35391_g1 | 0.836567658 | 0.20288 | N48 vs N46 |
| c35458_g2 | 0.15295087 | 0.0011428 | N48 vs N46 |
| c35511_g1 | 1.800946195 | 0.026444 | N48 vs N46 |
| c36174_g1 | 0.000643194 | 6.89E-21 | N48 vs N46 |
| c36434_g1 | 1.621215585 | 0.0000342 | N48 vs N46 |
| c36485_g1 | 1.940539016 | 0.0000788 | N48 vs N46 |
| c36499_g2 | 0.507953685 | 0.0000406 | N48 vs N46 |
| c36689_g1 | 4.10266596 | 8.98E-12 | N48 vs N46 |
| c36778_g3 | 0.010443338 | 0.00042105 | N48 vs N46 |
| c36863_g1 | 0.059722685 | 0.000000862 | N48 vs N46 |
| c36957_g4 | 61.49618607 | 8.26E-15 | N48 vs N46 |
| c37211_g1 | 0.538963744 | 0.00000564 | N48 vs N46 |
| c37446_g5 | 1.537912109 | 0.00015137 | N48 vs N46 |
| c37460_g3 | 0.625866686 | 0.43035 | N48 vs N46 |
| c51868_g1 | 0 | 1 | N48 vs N46 |

**Table S9 List of different GO terms**

**Table S10 List of different KEGG pathways**

**Table S11 All Primers used in quantitative Real Time-PCR. (A) Primer sequences for amplification of miRNAs. (B) Primer sequences for amplification of the targets of miRNAs.**

| A |  |
| --- | --- |
| **miRNA name** | **Sequence** |
| ahy-miR156a | TTGACAGAAGAGAGAGAGCAC |
| ama-miR396-3p | AAGCTCAAGAAAGCTGTGGGA |
| ata-miR395b-3p | CCAAGTGTTTGGGGGAACTC |
| ath-miR159b-3p | CTTTGGATTGAAGGGAGCTCTT |
| ath-miR159c | TTTGGATTGAAGGGAGCTCCT |
| ath-miR395a | TCTGAAGTGTTTGGGGGAACT |
| ath-miR396b-3p | TGCTCAAGAAAGCTGTGGGAA |
| ath-miR8175 | TTGATCCCCGGCAACGGC |
| bdi-miR159a-3p | GCTTGGATTGAAGGGAGCTC |
| cca-miR6113 | GTCTGAAACTCAAGAACACGTTG |
| gma-miR396a-3p | GGTTCAATAAAGCTGTGGGAAG |
| hci-miR156a | GTGACAGAAGAGAGTGAGTAC |
| lus-miR159b | TTTGGATTGAAGGGAGCTCTC |
| mtr-miR395a | ATGAAGTGTTTGGGGGAACTC |
| mtr-miR5286b | ACAAACTGGAGGCAAGGGAC |
| mtr-miR5290 | GAATTTGGAGAGAGATAGACACA |
| mtr-miR5559-5p | GGTACTTGGTGAATTGTTGGATC |
| mtr-miR5561-3p | CGGTCTATCTCTCTCTAAATGG |
| osa-miR159d | TTATTGGATTGAAGGGAGCTCC |
| osa-miR398b | TTAATGTGTTCTCAGGTCGCC |
| ppe-miR398b | AACGTGTTCTCAGGTCGCC |
| ppt-miR319a | CTTGGACTGAAGGGAGCTC |
| pta-miR159c | CTTGGATTGAAGGGAGCTCC |
| pta-miR319 | TTGGACTGAAGGGAGCTCC |
| ptc-miR171c | TTAGATTGAGCCGCGCCAATAT |
| sof-miR159c | CTTGGATTGAAGGGAGCTCC |
| stu-miR156f-5p | GCTGACAGAAGAGAGTGAGC |
| tae-miR395b | TTGAAGTGTTTGGGGGAACTC |
| B |  |
| **Gene name** | **Sequence** |
| c10498_g1 | F: TCGTCCAGGAGGAGAGGAA |
|  | R: CCCAACCAGCCAAATTAGCA |
| c14529_g1 | F: GCACATTGGACCCTTGACTG |
|  | R: GCTTCTCTTCGCTGACCAAC |
| c18626_g1 | F: GCCAACAACTCGCCACAAG |
|  | R: GATTTGACCTTACCACCACCAG |
| c20788_g1 | F: GGATGTGTCTGTGATGTTGGG |
|  | R: CCTTTCCTCTCCTCACGGTTA |
| c24424_g1 | F: TGGCACTTGACTTGGAATGG |
|  | R: GGAGTAGGTGGACTTGTGATTG |
| c27695_g1 | F: AGCACCAGCACCAACTACA |
|  | R: TAAGCAGCAGCACCATTAGC |
| c29588_g1 | F: AACCTGTTCGGCTTCCATTG |
|  | R: CGTATGCTGTGACATCCTCTC |
| c29993_g1 | F: CAGCTTTCTGTTTGCCGTCA |
|  | R: GGACTTCAACAGCCACATGAG |
| c30493_g1 | F: GGCGTTCCTCCATCTGTTATC |
|  | R: CCGTTGGTGTCTGAAGAGTG |
| c30598_g2 | F: GATATGAGCGAGCAAGAAGAGG |
|  | R: TGGAATGCGACCTGCTATCA |
| c32079_g1 | F: CCGCCTCTTCTTCCACGATT |
|  | R: CATCACGCTCTGCCTTGTTG |
| c33771_g1 | F: TGATGGAACACGTTGGTCTG |
|  | R: AGAAGTGCCATGCCTCTGT |
| c33813_g2 | F: GGTGGCTTCTTGACGGTTAC |
|  | R: CCTTCTACCAACGCATCTGTC |
| c33816_g1 | F: CCTTGTCCTCCTCTTGTTCGT |
|  | R: GTCCATGACTCACCGATACCA |
| c34206_g4 | F: GGAGAAGCTATGAGGATGATGG |
|  | R: TCAATCCAACCCGTGAAAGG |
| c34784_g1 | F: TGCGGTGGCGTTAAGAATC |
|  | R: GGAGACACTATGTGGACAAGC |
| c35097_g1 | F: GTCAGAGCCATCAGCATCATAG |
|  | R: GCTTCAGCTAGAGACCTACTCA |
| c35242_g1 | F: GTTCTTGTGGCATAGGTAGGAG |
|  | R: CCTGGTGGCTTAGTTGGAGT |
| c35391_g1 | F: AAGACTCTCCTCCACTCTGAAG |
|  | R: TGTGTTCTGAGCAAGGGTGA |
| c35458_g2 | F: CCATACACAGTGGCTGCTCTT |
|  | R: CAAGACCTGGTTGAGTGCTCTA |
| c35511_g1 | F: CGTTCTTCTTTACCGCAGAC |
|  | R: GCCACTCTTGATGCTGTAGA |
| c36174_g1 | F: CGAAAGGTTGGCGGGTCAT |
|  | R: CCGAATGGCATCCTCCTGTAA |
| c36434_g1 | F: GGCGTTGTCCATTGGTGAT |
|  | R: GTGAGTTCTCCTTGCGTCAA |
| c36485_g1 | F: GGAATGGATTGTCTTGGATGGC |
|  | R: AGCTTACCAGCAAGGAGCAT |
| c36499_g2 | F: AGTCTGGGAAGTCGGAAACG |
|  | R: CAGTCTCTTGCCATTGGTCATC |
| c36689_g1 | F: CACTGCACGAGCATCCTTC |
|  | R: GGCGGTTTGGACAGAGAGA |
| c36778_g3 | F: TGCTCTCCTATTCCGACTTCC |
|  | R: CGCCGTGACCTTCTCTTGA |
| c36863_g1 | F: GAAGGTCCACAGGTTCCATTCT |
|  | R: GCTGCGGTCAGTCAGTTCA |
| c36957_g4 | F; GAGGAGAAGACACTGGCAAGA |
|  | R: GTCGGTTCCCTTGTGAAGTTG |
| c37211_g1 | F: CAAAGCCCTTGCATCATCCA |
|  | R: GGAAGCGACTGAGTTTGTAGG |
| c37446_g5 | F: TCCGCTATGAACTGTCTCCTAG |
|  | R: ACTCAACCACAACGCTATGC |
| c37460_g3 | F: CGACGTGTTCCTGAGTTTCA |
|  | R: GCTCGCAATAGTGAGGTTGA |
| c51868_g1 | F: GCAAACAACAACCCGAAAGG |
|  | R: GGTCTCGAAGACGATGATGAC |

**Table S12** **a.The correlation of miRNAs and their target genes in N48vsN46**

| **miRNA** | **N48** | **N46** | **log2(N48/N46)** | **p-value** | **Targeted mRNA** | **N48** | **N46** | **log2(N48/N46)** | **p-value** | **miRNA-target correlation** |
| --- | --- | --- | --- | --- | --- | --- | --- | --- | --- | --- |
| lus-miR159b | 13391 | 28014.3 | -1.06485 | 0 | c36485_g1 | 253.943 | 130.8621 | 0.9564574 | 7.88E-05 | negative |
| ath-miR159c | 2772.2 | 7082.59 | -1.35327 | 0 | c36485_g1 | 253.943 | 130.8621 | 0.9564574 | 7.88E-05 | negative |
| hci-miR156a | 48.996 | 150.994 | -1.62376 | 9.5E-14 | c33813_g2 | 1779.62 | 1171.734 | 0.6029245 | 0.00058 | negative |
| ahy-miR156a | 10.651 | 38.1632 | -1.84117 | 4.4E-05 | c36689_g1 | 178.132 | 43.41862 | 2.0365617 | 8.98E-12 | negative |
| ahy-miR156a | 10.651 | 38.1632 | -1.84117 | 4.4E-05 | c33337_g3 | 79.7081 | 8.051497 | 3.3073973 | 3.91E-07 | negative |
| mtr-miR5261 | 0 | 25.7187 | Inf | 1.2E-07 | c36957_g4 | 54.0382 | 0.878725 | 5.942425 | 8.26E-15 | negative |
| lus-miR159b | 13391 | 28014.3 | -1.06485 | 0 | c30493_g1 | 0.34813 | 261.0828 | -9.550669 | 1.69E-05 | positive |
| ath-miR159b-3p | 13816 | 29204.8 | -1.07986 | 0 | c30493_g1 | 0.34813 | 261.0828 | -9.550669 | 1.69E-05 | positive |
| ath-miR159c | 2772.2 | 7082.59 | -1.35327 | 0 | c35097_g1 | 0 | 139.2881 | Inf | 1.35E-37 | positive |
| stu-miR156f-5p | 1332.8 | 3696.85 | -1.47181 | 8E-259 | c20788_g1 | 0 | 16.76707 | Inf | 7.92E-06 | positive |
| osa-miR159c | 9.9411 | 29.0372 | -1.54642 | 0.00161 | c30493_g1 | 0.34813 | 261.0828 | -9.550669 | 1.69E-05 | positive |
| pta-miR319 | 154.8 | 336.832 | -1.12165 | 3.8E-17 | c30493_g1 | 0.34813 | 261.0828 | -9.550669 | 1.69E-05 | positive |
| ath-miR159c | 2772.2 | 7082.59 | -1.35327 | 0 | c30493_g1 | 0.34813 | 261.0828 | -9.550669 | 1.69E-05 | positive |
| ppt-miR319a | 149.83 | 334.343 | -1.15803 | 7.94E-18 | c35097_g1 | 0 | 139.2881 | Inf | 1.35E-37 | positive |
| hci-miR156a | 48.996 | 150.994 | -1.62376 | 9.5E-14 | c20788_g1 | 0 | 16.76707 | Inf | 7.92E-06 | positive |
| osa-miR159d | 5.6806 | 19.9112 | -1.80946 | 0.00355 | c30493_g1 | 0.34813 | 261.0828 | -9.550669 | 1.69E-05 | positive |
| pta-miR319 | 154.8 | 336.832 | -1.12165 | 3.8E-17 | c35097_g1 | 0 | 139.2881 | Inf | 1.35E-37 | positive |
| mtr-miR5261 | 0 | 25.7187 | Inf | 1.2E-07 | c10498_g1 | 0 | 36.0557 | Inf | 1.22E-11 | positive |
| mtr-miR395a | 198.11 | 12.4445 | 3.99274 | 5.9E-43 | c36499_g2 | 151.76 | 298.7679 | -0.977231 | 4.06E-05 | negative |
| tae-miR395b | 159.77 | 12.4445 | 3.6824 | 3.4E-33 | c36499_g2 | 151.76 | 298.7679 | -0.977231 | 4.06E-05 | negative |
| tae-miR395b | 159.77 | 12.4445 | 3.6824 | 3.4E-33 | c37446_g5 | 7329.06 | 4765.589 | 0.6209731 | 0.000151 | positive |
| ata-miR395b-3p | 159.77 | 11.6149 | 3.78193 | 8.38E-34 | c24424_g1 | 16.9638 | 0.258147 | 6.0381213 | 1.62E-05 | positive |
| ath-miR395a | 34.084 | 4.14817 | 3.03854 | 3.21E-07 | c37446_g5 | 7329.06 | 4765.589 | 0.6209731 | 0.000151 | positive |
| ata-miR395b-3p | 159.77 | 11.6149 | 3.78193 | 8.4E-34 | c37484_g3 | 95.9869 | 0 | Inf | 2.53E-29 | positive |

**Table S12 a.The correlation of miRNAs and their target genes in N49vsN47**

| **miRNA** | **N49** | **N47** | **log2(N49/N47)** | **p-value** | **Targeted mRNA** | **N49** | **N47** | **log2(N49/N47)** | **p-value** | **miRNA-target correlation** |
| --- | --- | --- | --- | --- | --- | --- | --- | --- | --- | --- |
| mtr-miR5261 | 0 | 15.4147 | -4.946 | 4.01E-05 | c36957_g4 | 40.6521 | 0.654311 | 5.9572 | 9.50E-17 | negative |
| cca-miR6113 | 0 | 9.90944 | -3.454 | 0.00271 | c36957_g4 | 40.6521 | 0.654311 | 5.9572 | 9.50E-17 | negative |
| cca-miR6113 | 0 | 9.90944 | -3.454 | 0.00271 | c37460_g3 | 11.7541 | 0.352603 | 5.059 | 1.41E-05 | negative |
| ath-miR159c | 2406.6 | 6419.12 | -1.415 | 0 | c30493_g1 | 1.65758 | 151.4728 | -6.5138 | 8.27E-12 | positive |
| stu-miR156f-5p | 892.31 | 2651.88 | -1.571 | 5.16E-222 | c20788_g1 | 0 | 15.27179 | Inf | 4.41E-08 | positive |
| osa-miR398b | 666.3 | 1806.27 | -1.439 | 1.06E-134 | c27695_g1 | 198.697 | 384.1936 | -0.9513 | 0.000285 | positive |
| pta-miR319 | 90.407 | 334.719 | -1.888 | 3.59E-37 | c35097_g1 | 0.33962 | 125.0382 | -8.5242 | 3.41E-45 | positive |
| ppt-miR319a | 85.886 | 331.966 | -1.951 | 2.47E-38 | c35097_g1 | 0.33962 | 125.0382 | -8.5242 | 3.41E-45 | positive |
| bdi-miR159a-3p | 52.436 | 126.07 | -1.266 | 2.59E-09 | c30493_g1 | 1.65758 | 151.4728 | -6.5138 | 8.27E-12 | positive |
| mtr-miR5261 | 0 | 15.4147 | -4.946 | 4.01E-05 | c34232_g1 | 116.767 | 292.2657 | -1.3236 | 1.11E-08 | positive |
| mtr-miR5261 | 0 | 15.4147 | -4.946 | 4.01E-05 | c10498_g1 | 0 | 25.93683 | Inf | 2.76E-11 | positive |
| ath-miR159c | 2406.6 | 6419.12 | -1.415 | 0 | c35097_g1 | 0.33962 | 125.0382 | -8.5242 | 3.41E-45 | positive |
| pta-miR319 | 90.407 | 334.719 | -1.888 | 3.59E-37 | c30493_g1 | 1.65758 | 151.4728 | -6.5138 | 8.27E-12 | positive |
| hci-miR156a | 37.067 | 132.676 | -1.84 | 3.07E-15 | c20788_g1 | 0 | 15.27179 | Inf | 4.41E-08 | positive |
| gma-miR171n | 15.369 | 37.4357 | -1.284 | 0.001039 | c34656_g1 | 68.5496 | 150.1651 | -1.1313 | 1.05E-05 | positive |
| ppe-miR398b | 0 | 11.561 | -3.677 | 0.000924 | c27695_g1 | 198.697 | 384.1936 | -0.9513 | 0.000285 | positive |
| mtr-miR395a | 59.668 | 27.5262 | 1.1162 | 0.001244 | c36499_g2 | 147.492 | 274.2578 | -0.8949 | 8.41E-05 | negative |
| tae-miR395b | 50.628 | 23.122 | 1.1307 | 0.002628 | c36499_g2 | 147.492 | 274.2578 | -0.8949 | 8.41E-05 | negative |
| gma-miR5368 | 100.35 | 41.2893 | 1.2812 | 2.38E-06 | c18626_g1 | 97.0897 | 0.352603 | 8.1051 | 5.49E-37 | positive |
| ata-miR395b-3p | 50.628 | 23.122 | 1.1307 | 0.002628 | c24424_g1 | 10.7757 | 0.705206 | 3.9336 | 0.000153 | positive |
| ata-miR395b-3p | 50.628 | 23.122 | 1.1307 | 0.002628 | c37484_g3 | 77.7859 | 0 | Inf | 1.81E-31 | positive |
| ath-miR8175 | 25.314 | 4.95472 | 2.3531 | 0.000194 | c36174_g1 | 2408.34 | 0.654311 | 11.846 | 1.90E-10 | positive |
